# Fibrin defines tissue stiffness and biomechanical signaling in regenerating zebrafish hearts as revealed by high-resolution stiffness mapping

**DOI:** 10.1016/j.isci.2026.115231

**Published:** 2026-03-04

**Authors:** Juliane Münch, Tuli Pramanik, Isabell Tunn, Leona Simon, Claudia Jasmin Rödel, Shahrouz Amini, Peter Fratzl, Kerstin Blank, Ondine Cleaver, Salim Abdelilah-Seyfried

**Affiliations:** 1 Institute of Biochemistry and Biology, University of Potsdam, 14476 Potsdam, Germany; 2 Department of Molecular Biology and Center for Regenerative Science and Medicine, University of Texas Southwestern Medical Center, Dallas, TX 75390, USA; 3 Fraunhofer Institute for Applied Polymer Research, 14476 Potsdam, Germany; 4 Department of Biomaterials, Max Planck Institute of Colloids and Interfaces, 14476 Potsdam, Germany; 5 Mechano(bio)chemistry, Max Planck Institute of Colloids and Interfaces, 14476 Potsdam, Germany; 6 Institute of Experimental Physics, Johannes Kepler University Linz, 4040 Linz, Austria

**Keywords:** Cardiovascular medicine, Mechanobiology, Biological sciences, Tissue engineering

## Abstract

Myocardial infarction in humans causes an irreversible scar, which permanently impairs cardiac mechanical properties and physiological functions. The zebrafish heart resolves scar tissue and regenerates injured myocardium. To study mechanical properties during regeneration, we developed a method combining atomic force microscope-based nanoindentation with confocal microscopy and generated a high-resolution elasticity map of the zebrafish heart. This revealed distinct regions of stiffness within the injury site, including a stiff area that is cell-poor and fibrin-rich, contrasting with the softer injury center and surrounding myocardium. Whole-transcriptome analyses uncovered several components of the coagulation and fibrinolysis cascades in the regenerating heart. Pharmacological inhibition of the fibrinolysis regulator Serpine1 demonstrated that reduced fibrin-mediated stiffness impacts the biomechanical Hippo pathway in adjacent endocardial cells. Our approach characterizes the mechanical properties of different regions in the regenerating heart and shows that the biomechanical environment and mechanotransductive signaling are crucial components for understanding regenerative mechanisms.

## Highlights

• The regenerating zebrafish heart presents regions with different mechanical properties
• A cell-poor fibrin deposit is the stiffest compartment at the injury site
• Serpine1-inhibition softens the fibrin deposit
• Softer fibrin changes endocardial/endothelial Yap-signalling and regenerative processes

## Graphical abstract

**Figure:**
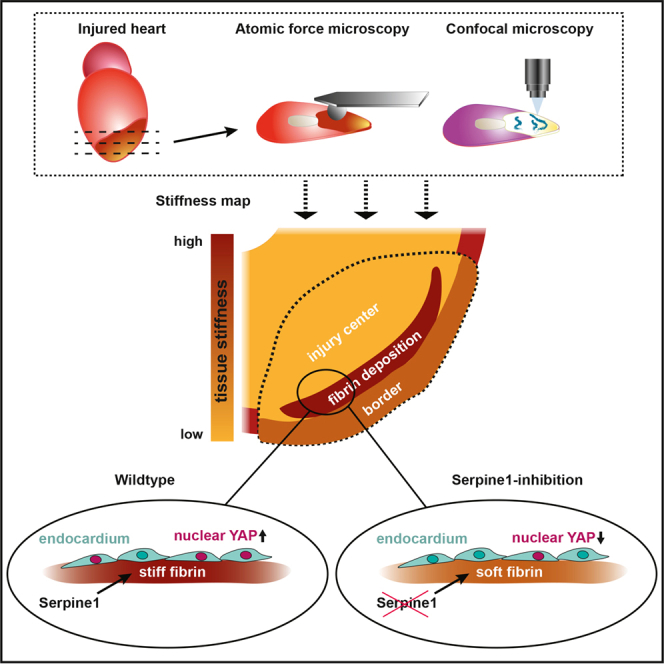

## Introduction

The heart is a mechanically active organ that constantly resists mechanical load while requiring adequate compliance for its blood-pumping function. These physiological functions are impaired when cardiac injuries and infarction lead to a loss of cardiac muscle. In such cases, the myocardium is permanently replaced by fibrotic scar tissue, which contains extracellular matrix (ECM) depositions and cardiac fibroblasts.^1^^,^^2^ This replacement preserves the structural integrity of the ventricle and protects against ventricular rupture and dilative remodeling.^3^ However, these changes in tissue composition affect the mechanical properties of the cardiac ventricle,^4^ with consequences for the heart’s diastolic function.^3^ On the cellular level, the physical properties of the environment also influence cell behaviors, including cell migration, proliferation, and differentiation.^2^^,^^5^ This can potentially hinder or promote healing processes in diseased hearts. While there is extensive knowledge about beneficial cardiac reparative processes from models that regenerate the heart, such as zebrafish, salamander, or the neonatal mouse,^6^^,^^7^^,^^8^^,^^9^ the impact of changes in mechanical properties on healing processes in regenerating hearts remains largely unknown. Understanding both biochemical and biomechanical factors is crucial for developing effective cardiac regenerative therapies.

The mechanical properties of biological samples have been studied using different approaches, including atomic force microscopy (AFM)-based nanoindentation, micropipette aspiration, ultrasound elastography, and Brillouin microscopy.^10^^,^^11^^,^^12^^,^^13^^,^^14^ Tissue stiffness is quantified using the Young's modulus, which describes the force per area (stress) needed to deform an elastic material. In mammals, heart stiffness ranges from 18 ± 2 kPa in healthy tissue to as high as 55 ± 15 kPa in infarcted regions.^4^ Changes in stiffness can result from alterations to intracellular components, such as the sarcomeric protein TITIN or cytoskeleton elements such as microtubules, or modifications to the extracellular matrix (ECM).^15^

Biomechanical forces impacting tissues adjacent to cardiomyocytes are sensed by membrane-bound mechanosensory complexes, which convert these forces into biochemical signals and cytoskeletal modifications through a process known as mechanotransduction. This mechanosensing and force transmission machinery includes mechanosensitive ion channels of the Piezo or transient receptor potential cation Trpv families.^16^^,^^17^^,^^18^ Activation of these channels triggers downstream biomechanical signaling through various pathways, including the Hippo pathway.

Biomechanical signaling via the Hippo pathway^19^^,^^20^ involves a phosphorylation cascade that leads to the degradation or cytoplasmic retention of Yes-associated protein (Yap) and the WW-domain-containing transcription regulator 1 (Taz).^21^ When Hippo pathway signaling is inactive, Yap and Taz translocate to the nucleus, where they activate target genes.^21^^,^^22^^,^^23^ Activation of the Hippo pathway involves mechanical signals, ECM stiffness, and cell-cell contact inhibition^24^^,^^25^ and regulates reparative and regenerative processes in various organs, including the heart.^22^^,^^26^ In the mammalian heart, active Hippo signaling inhibits cardiomyocyte renewal. Conversely, constitutively active YAP supports cardiac regeneration by initiating an embryonic and proliferative gene program.^27^^,^^28^

Unlike mammals, the zebrafish heart completely regenerates after ventricular resection, cryoinjury, or cardiomyocyte ablation.^29^^,^^30^^,^^31^^,^^32^^,^^33^ Following cryoinjury, the heart undergoes an initial inflammatory response phase where a fibrin-rich network is deposited throughout the wound site. This network harbors immune cells, which are recruited to clear dead cells and debris.^30^ The formation of this fibrin-fiber network is a common injury response, resulting from the coagulation cascade that transforms fibrinogen into fibrin.^34^

During the subsequent reparative phase, fibrin deposits become more restricted to the injury site’s periphery. Various cell types, such as fibroblasts, macrophages, and endocardial cells, secrete ECM molecules such as collagen, fibronectin, hyaluronic acid, and Tenascin C. These molecules form a scar that temporarily replaces lost cardiac muscle,^30^^,^^35^^,^^36^^,^^37^^,^^38^ resembling fibrosis in the injured human heart.

Simultaneously, cells adjacent to the injury site become activated, dedifferentiate, and proliferate. This includes cells from the myocardium, epicardium, and endocardium.^39^^,^^40^ Finally, newly formed and vasculated myocardium replaces the fibrotic tissue, and the scar tissue resolves within 60–90 days,^35^^,^^36^^,^^41^^,^^42^ While studies on scar resolution in zebrafish have largely focused on collagen deposits,^36^ the dynamics and function of fibrin networks in the injured heart have not been previously explored. Zebrafish serve as an excellent model for studying fibrin clot formation and lysis since coagulation proteins are functionally conserved between fish and mammals.^43^^,^^44^

Our understanding of the physical changes occurring within the regenerating zebrafish cardiac ventricle is limited. Specifically, it remains unclear whether biomechanical pathway signaling influences regenerative processes in the injured adult zebrafish ventricle. Studies measuring the mechanical properties of the cardiac ECM after the decellularization of resected zebrafish hearts have shown a decrease in stiffness, coinciding with shifts in ECM protein composition.^45^ Further research has identified the biomechanical Hippo pathway as playing a role in scar formation, ECM deposition, macrophage infiltration, and myocardial proliferation.^46^^,^^47^ Remarkably, the loss of caveolae - plasma membrane invaginations that act as mechanosensors and transducers^48^-leads to reduced ventricular stiffness and impaired regeneration.^49^ Similarly, in zebrafish axonal regeneration, the significance of the ECM for neuron regenerative capacity has been well-documented.^10^^,^^50^ Despite these studies, a detailed spatial and temporal characterization of the mechanical properties of injured zebrafish cardiac tissues is lacking. Such characterization would enhance our understanding of how transiently formed tissue, such as scars, alters mechanotransductive signaling during regeneration.

In this study, we introduce a novel methodology for creating a finely gridded stiffness map of the regenerating zebrafish heart. This approach revealed that scar deposition resulting from cryoinjury does not alter the overall, global mechanical properties of the ventricle. However, detailed AFM stiffness measurements at different regeneration stages identified a cell-poor, fibrin-rich region as the stiffest area, while the center of the injury displayed mechanical properties similar to the adjacent myocardium.

Environmental scanning electron microscopy (ESEM) further revealed a dense, sponge-like morphology in fibrin deposits. Additionally, gene expression analyses detected several key factors involved in fibrin deposition and lysis during different hallmark stages of zebrafish ventricular regeneration. Conducting functional experiments, we identified the fibrinolysis regulator Serpine1 as a crucial determinant of the stiffness of fibrin deposits. Our findings show that changes in stiffness due to Serpine1 influence Hippo signaling in endocardial cells adjacent to fibrin deposits, potentially modifying pro-regenerative signaling. Overall, we characterized the global and local mechanical properties of injured cardiac tissue in zebrafish with unmatched resolution, identifying fibrin as marking the stiffest compartment. The discovery that fibrin clots affect the mechanical properties of injured tissue - and consequently biomechanical regenerative signaling - suggests new potential for fibrin-based scaffolds in regenerative therapies.

## Results

### Global mechanical properties of the injured zebrafish heart

**Figure 1:**
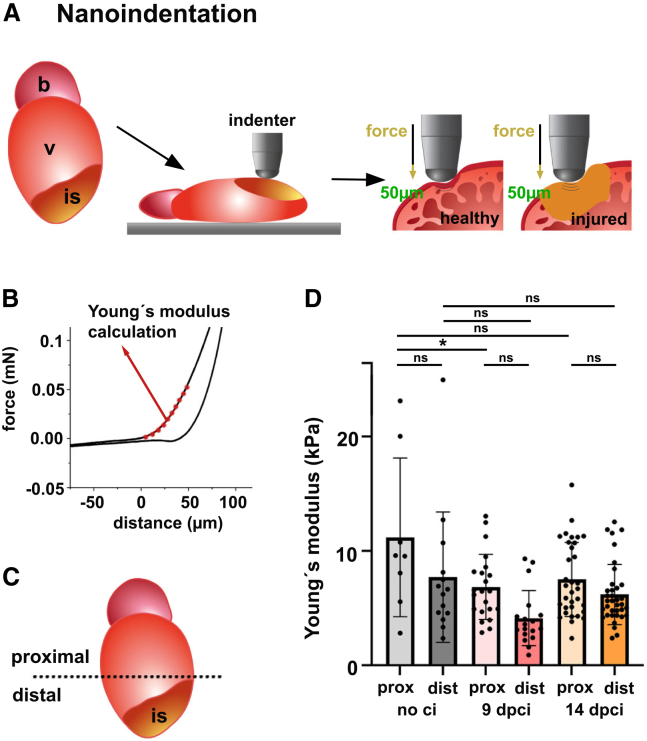
Large-displacement depth-sensing nanoidentation approach on whole hearts (A) Scheme indicating the injured zebrafish heart and its position together with the cono-spherical indenter (r = 100 μm) during measurements. Whole hearts were measured. Left panels show the depth of indentation (50 μm) in healthy and injured hearts. b-bulbus arteriosus, v-ventricle, is-injury site. (B) Example of force-distance curve indicating the region (red), which was used for Young's modulus calculations. (C) Scheme indicating proximal (>300 μm from the apex) and distal region (0-300 μm from the apex) of the heart. is-injury site. (D) Graph showing Young's modulus values of proximal (prox) and distal (dist) regions of non-injured (no ci), 9 and 14 days post cryoinjury (dpi). Individual measurements and means (bars) +/− SD are represented. One-way ANOVA with Tukey’s multiple comparison test did not reveal significant differences between proximal and distal region within one timepoint. ns: not significant, ∗*p* = 0.046. See also Figure S1.

We developed a large-displacement depth-sensing nanoindentation protocol to study the global mechanical properties of the zebrafish heart both in homeostasis and after cryoinjury.^51^ This method uses a rigid cono-spherical probe with a radius of 100 μm to indent freshly dissected hearts in various positions under hydrated conditions (Figure 1A). The vertical displacement of the probe and force during loading (extent) and unloading (retraction) are recorded to generate a force-distance curve (Figure 1B). Using the Hertzian contact model, commonly applied in nanoindentation studies with spherical indenters, we calculated the indentation elastic modulus to describe tissue stiffness^52^^,^^53^ (see STAR Methods).

For these calculations, we focused on a section of the extended curve, from 0 to ≤50 μm (Figure 1B, red), indicating the distance the tip indented into the tissue (Figure 1A). This allowed us to quantify the mechanical properties of different cardiac regions, including the outer epicardium, cortical myocardium (with >20 μm thickness),^54^ the thin underlying primordial myocardium, and peripheral areas of the trabeculated myocardium in non-injured hearts. For injured hearts, we analyzed corresponding regions of the injury site (Figure 1A).

Although the nanoindenter was equipped with a microscope, it did not facilitate the precise localization of the injury site. Cryoinjury predominantly affects the apical distal part of the ventricle (Figure 1C), leading us to separately analyze proximal and distal regions, calculating the indentation elastic modulus for both in injured versus non-injured hearts. In non-injured hearts, global stiffness was similar across distal and proximal regions, with an indentation elastic modulus of 11.2 kPa (±7) and 7.7 kPa (±6), respectively (Figure 1D).

Injury assessments were conducted at 9 and 14 days post-cryoinjury (dpci). These time points were chosen based on the closure of the wound by an epicardial sheet at 9 dpci^55^ and migration of cortical myocardial cells over the wound site by 14 dpci.^54^ Injured hearts showed similar tissue stiffness (Figure 1D) and Young’s modulus in both regions at 9 dpci (6.7 ± 3 kPa and 4.1 ± 2 kPa) and at 14 dpci (7.5 ± 3 kPa and 6.2 ± 3 kPa). Large-displacement depth-sensing nanoindentation measurements revealed variations of Young's modulus ranging from 4 to 11 kPa, with no significant differences between injured and healthy tissues. This suggests that changes in tissue composition in the injured zebrafish heart do not affect its global mechanical properties, or variations are too subtle to be detected by this method.

### A high-resolution elasticity map reveals differences between the cortical myocardium and injury sites

**Figure 2:**
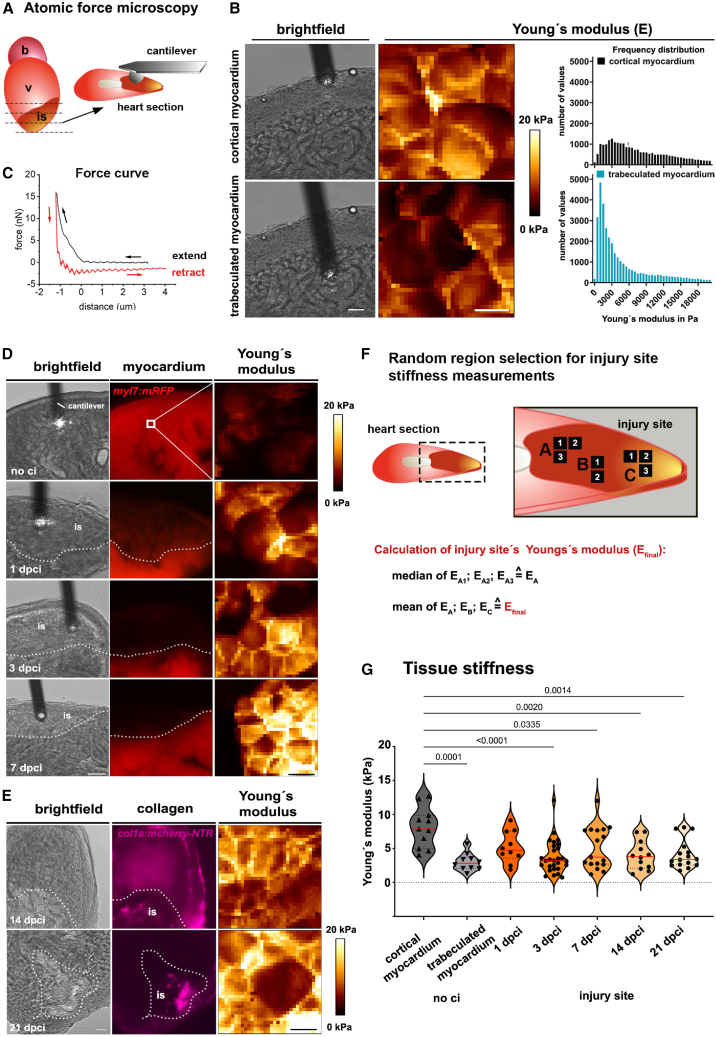
Atomic force microscopy (AFM) on heart sections reveals stiffness differences between cortical myocardium, trabeculated myocardium, and the injury site (A) Scheme indicating the AFM approach. For measurements, 100 μm thick heart sections from freshly dissected hearts were used. A spherical tip (r = 5.4 μm) indented on different regions of the sections. (B) Brightfield images showing the cantilever indenting the compact myocardium (upper panel) and the trabeculated myocardium (lower panel). Maps represent Young's modulus stiffness values of 20 × 20 μm. Frequency distribution graphs show representative Young's modulus values of the cortical (black) and the trabeculated (turquoise) myocardium of one heart. (C) Example force-distance curve from one AFM measurement, indicating retract and extend curve. (D) Brightfield image indicating the cantilever position and corresponding fluorescence image of heart sections from *Tg(myl7:mRFP)*^*md3*^, myocardial reporter fish. The lack of *myl7:mRFP* (1, 3, 7 dpci) indicates the injury site (is), dotted lines demarcate the inner injury border. Corresponding color maps show Young's modulus values (0-20 kPa) of one measurement. (E) Brightfield and corresponding fluorescence image of *Tg(col1a2:LoxP-mcherry-NTR)*^*cn11*^ fish hearts at 14 and 21 dpci. Mcherry^+^- cells are present at the injury site (is), demarcated by the dotted line. Corresponding color maps show Young's modulus values (0-20 kPa) of one measurement. (F) Scheme showing calculations of Young's modulus for one injury site: 2 to 3 stiffness maps were recorded in regions A, B, and C. The median (E_A_, E_B,_ or E_C_) of all values from those maps of one region was calculated. The mean of the medians of each region represents the Young's modulus of one injury site (E_final_). (G) Violin plot representing Young's modulus values of cortical and trabeculated myocardium from non-injured hearts and from injury sites of 1, 3, 7, 14, and 21 dpci hearts. Individual measurements and medians (red lines) are represented., One-way ANOVA with Tukey’s multiple comparison test results are indicated. F-test's *p*-values compared to trabeculated myocardium for 1dpci: 0.091, 3dpci: 0.052, 7dpci: 0.011, 14 dpci: 0.138, 2dpci: 0.132, cortical myocardium: 0.021. Scale bars, 50 μm in B, D, and E left panels, 5 μm in B, D, and E right panels.

Tissue stiffness in a cryoinjured heart may vary on a finer scale than can be assessed using global nanoindentation techniques. Therefore, we sought a method to investigate the stiffness of injured and non-injured cardiac tissue with higher resolution. We developed another nanoindentation approach utilizing AFM equipped with a colloidal tip measuring 10.8 μm in width for microscale tissue probing in zebrafish cardiac tissue. This AFM technique was combined with fluorescence microscopy to identify injured and healthy tissue in transgenic zebrafish, which carry cardiac tissue reporters. Previously, tissue stiffness measurements were based on the ECM of decellularized injured zebrafish hearts.^45^ Our approach aimed to measure the mechanical properties of tissue within the biomechanical environment of injury sites. To avoid alterations in tissue properties that may occur during fixation, we used freshly dissected hearts, which were vibratome-sectioned into 100 μm thick transverse sections (Figure 2A). This thickness was suitable for AFM nanoindentation because thinner sections are challenging to produce from freshly prepared cardiac tissue and thicker sections often mask the injury site. First, we used brightfield microscopy to localize the outer cortical and inner trabeculated myocardium on heart sections and measured the mechanical properties of both regions in the uninjured heart (Figure 2B). The spatial resolution of the stiffness map provided 32 × 32 data points in areas measuring 20 μm × 20 μm in different cortical or trabeculated regions of the myocardium (Figure 2B). Each indentation generated a force/distance curve (Figure 2C; Figure S1A). Extend curves were then fitted in a batch approach using the Hertzian contact model,^52^ and the Young’s modulus (E) was calculated as a value for tissue stiffness (Figure 2C; Figure S1, also see STAR Methods). The distribution of Young’s moduli revealed that the outer myocardial layer is stiffer than the inner trabeculated myocardium in the uninjured heart (Figure 2B).

To determine if the mechanical properties of injured tissue differ from those of uninjured cardiac tissue, we performed cryoinjury^56^ on Tg(*myl7:mRFP*)^*md3*^^57^ or Tg(*col1a2:loxP-mCherryNTR*)^*cn11*^^36^ cardiac reporter zebrafish. We identified the injury site on heart sections by the absence of *myl7*:mRFP^+^ cardiomyocytes at early stages (Figure 2D; 1–7 dpci) or the presence of *col1a2*:mCherry^+^ fibroblasts (Figure 2E; 14, 21 dpci) later in the regeneration process. AFM nanoindentation was subsequently performed on these sections, recording 2 to 5 stiffness maps with a resolution of 20 μm × 20 μm in various regions across the injury site (Figure 2F; regions A, B, C). To determine the Young’s modulus (E) representative of the entire injury site (Figure 2F; E_final_), we averaged the median stiffness values from different regions (Figures 2F and 2G). Comparing Young’s moduli showed that injury sites, at most time points (3, 7, 14, and 21 dpci), had stiffness values similar to those of trabeculated myocardium (3.0 ± 1.3 kPa) but significantly lower than cortical myocardium in non-injured hearts (8.1 ± 2.9 kPa) (Figure 2G). Consequently, the average stiffness of injured tissue during key regeneration stages reflects the mechanical properties of healthy trabeculated myocardium rather than cortical myocardium.

### High-resolution elasticity mapping detects unique mechanical properties of a decellularized injury region

**Figure 3:**
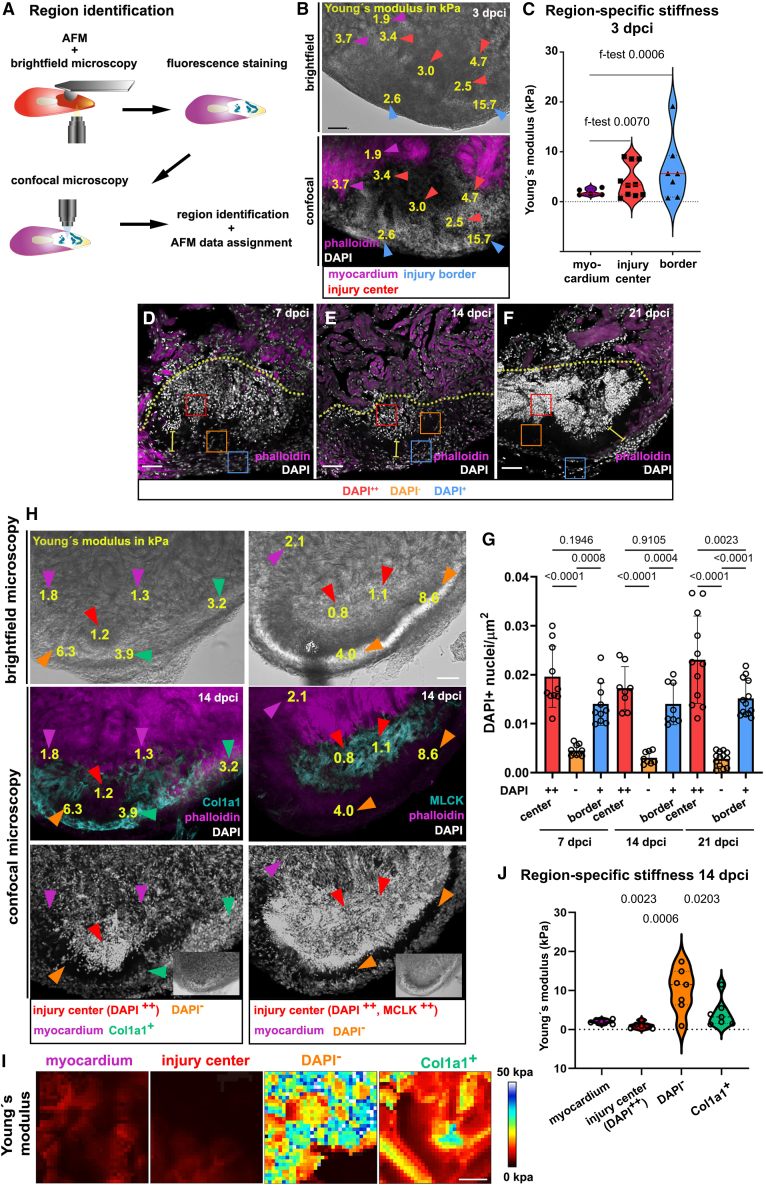
Region-specific AFM stiffness measurements (A) Scheme indicating the experimental approach. Following AFM and brightfield microscopy, fluorescence staining and confocal microscopy were performed. Different regions were identified, and images were compared to assign corresponding AFM data to each section. (B) Representative brightfield and confocal images of phalloidin and DAPI-stained injured hearts (3 dpci). Yellow numbers indicate the Young's modulus values measured at the corresponding positions. Arrows point to identified regions: purple myocardium; blue injury border; red injury center. (C) Violine plots showing individual and medians (red) of Young's modulus values from the myocardium, injury center, and border region (3 dpci). f-test results are shown. Red line = median. (D–F) Representative heart sections stained for DAPI and phalloidin. Squares indicate DAPI^++^ injury center (red), DAPI^−^-region (orange), and DAPI^+^ injury border (blue). Dotted lines demarcate the injury site. (G) Bar chart depicting DAPI^+^ content from heart sections 7, 14, and 21 dpci from different regions. Individual values and means (bars) +/− SD are represented. One-way ANOVA with Tukey’s multiple comparison test results are indicated. (H) Representative brightfield and confocal images of Col1a1 or Myosin light-chain kinase (MLCK), phalloidin, and DAPI. Yellow numbers depict the Young's modulus values in this region. Arrowheads indicated the regions: purple, myocardium; blue, Col1a1^+^ -region; red, injury center; orange, DAPI^−^ -region. (I) Color maps representative for each region, indicating AFM-measured Young's modulus (0-50 kPa) on a 20 × 20 μm field. (J) Violin plot showing individual and medians (red) of Young's modulus values of specific regions. One-way ANOVA with Tukey’s multiple comparison test results are indicated. Scale bars, 50 μm in B, D–F, H; 5 μm in I. See also Figure S2.

Although the average stiffness values of injured tissue were similar to those of non-injured trabecular myocardium, there was considerable variation within the dataset. Statistical analyses using an f-test hinted at regional differences within the 7 dpci injury site (Figure 2G). This prompted us to further characterize the mechanical properties of specific injury regions by combining AFM nanoindentation with high-resolution confocal microscopy. We measured the stiffness of different regions (4–8 per heart section) within sections of injured hearts at 3 and 7 dpci using AFM nanoindentation and acquired brightfield images of the region of interest (Figures 3A and 3B). Following AFM probing, heart sections were fixed for fluorescence labeling with DAPI and counter-labeled with rhodamine-phalloidin to stain the myocardium (Figure 3A). By comparing confocal images of stained sections with brightfield images, we assigned the AFM stiffness values to specific regions, such as the myocardium adjacent to the wound, the injury border, and the center of injury (indicated by arrowheads in Figure 3B). At 3 dpci, the average Young’s modulus of these three regions did not differ significantly (Figure 3C). However, the f-test demonstrated higher variability in stiffness values at the injury center and border zone compared to the homogeneous mechanical properties of the adjacent healthy myocardium. At 7 dpci, we recognized three distinct regions based on cell density. There was a cell-rich center of injury, as indicated by a high number of DAPI^+^ nuclei (DAPI^++^), an outer injury border (DAPI^+^) containing fewer cells, and a stripe-shaped compartment between these regions with few cells (DAPI^−^ in Figures 3D–3G). This regionalization became even more pronounced at 14 and 21 dpci (Figures 3D–3G), aligning with previous reports of increased organization at these stages.^30^^,^^58^

In mammalian hearts, increased stiffness is largely due to ECM depositions, such as collagens secreted by fibroblasts.^15^^,^^59^^,^^60^ At 7 and 14 dpci, the injury site in zebrafish also contains fibroblasts and various collagens.^35^^,^^36^ We combined DAPI staining with immunolabeling using antibodies against the ECM protein Collagen1a1 (Col1a1) and Myosin light-chain kinase (MLCK), which marks smooth muscle cells or ECM-producing myofibroblasts^55^ (Figure 3H).

Col1a1 marked areas at the injury border (DAPI^+^) and the center of injury (DAPI^++^) (Figure 3H; Figure S2A). MLCK predominantly marked the DAPI^++^ center of injury (Figure 3H), and there was a de-cellularized DAPI^−^ area largely devoid of Col1a1 or MLCK expression (Figure 3H). For stiffness analyses, we plotted AFM-measured Young’s moduli onto corresponding locations in brightfield images of heart sections (Figure 3H) and compared these with related confocal images of immunolabeled sections. This revealed similar mechanical properties in the myocardium adjacent to the injury site (2.1 ± 0.5 kPa) and the center of injury (1.0 ± 0.8 kPa) at 14 dpci (Figures 3I and 3J; Figure S2B). In contrast, the DAPI^−^ region was significantly stiffer (10.1 ± 5.6 kPa) than the other regions (Figure 3J). The f-test highlighted the heterogeneity in mechanical properties of both the DAPI^−^ region (f-test: 4.82 E^−05^) and the Col1a1^+^ region (f-test: 4.27 E^−04^), but not the injury center (f-test: 0.31). In summary, AFM-based high-resolution stiffness analyses unveiled a surprising heterogeneity in mechanical properties across different regions of the injury site. Tissue stiffness at the injury center was highly variable at 3 dpci but became more homogenous by 14 dpci when regions were defined by cell density. Notably, at this stage, a non-cellularized region constituted the stiffest compartment within the injured ventricle.

### The stiffest compartment at the injury site is a fibrin-rich scaffold

**Figure 4:**
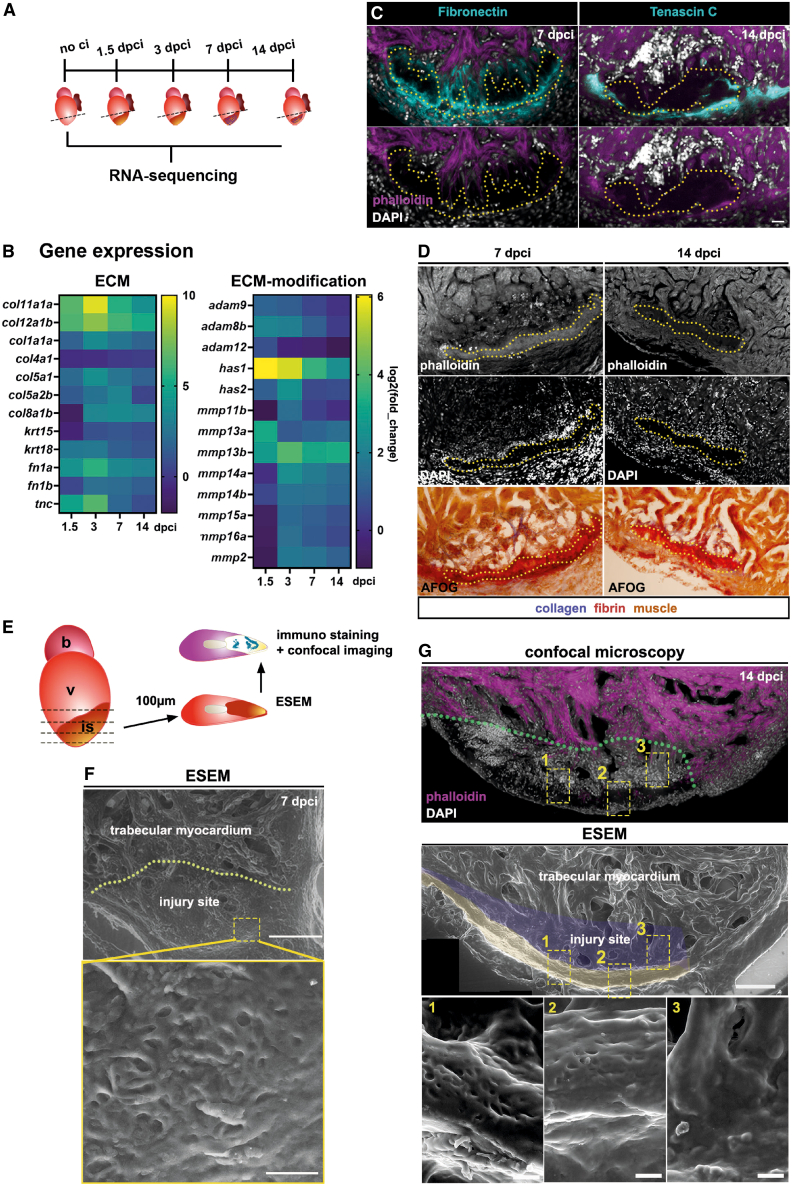
A Fibrin scaffold defines a DAPI^-^ -region at the injury site (A) Experimental approach for RNA-sequencing experiment, using apex tissue from different time points upon cryoinjury. (B) Heatmaps indicating log2 (fold-changes) of genes related to encoding extracellular matrix molecules (ECM) or ECM-mediators expressed at 1.5, 3, 7, 14 dpci. (C) Representative images from immune-stained heart sections with Fibronectin (left) and Tenascin-C (right) deposits at the injury site. The dotted line indicates the DAPI^−^ -region. (D) Representative images of acid-fuchsin-orange (AFOG)-stained heart sections (7, 14 dpci) that were previously stained for phalloidin and DAPI. Dotted lines indicate the DAPI^−^ -region, which coincides with fibrin deposits. (E) Scheme indicating the imaging approach: 100 μm-transversal heart sections were imaged using environmental scanning microscopy (ESEM). The same sections underwent fluorescence staining and confocal imaging. (F) Representative ESEM-image of the injury site (dotted line) at 7 dpci. The inset shows spongy fibrin deposits. (G) ESEM-image of a heart section (14 dpci) showing trabecular myocardium and injury site (blue, yellow). The dotted line demarcates the injury site. Inset 1 and 2 indicate spongy fibrin deposits (yellow), and inset 3 indicates regenerating myocardium. Image of DAPI^−^ and phalloidin staining shows that fibrin deposits coincide with the DAPI^−^ -region (arrowheads). The dotted line demarcates the injury site. Scale bars, 50 μm in F (upper panel), 10 μm in F and G (lower panels), 20 μm in C, D, and G upper panels. See also Figure S3.

To identify potential regulators that define mechanical properties in the zebrafish injured heart, we conducted bulk RNA sequencing of the injured apical region of the heart at 1.5, 3, 7, 14, and 21 dpci, comparing this data with non-injured cardiac tissue (Figure 4A). This analysis revealed the upregulation of genes encoding ECM proteins, including many collagens, fibronectins, keratins, and Tenascin-C (TenC), at both early and later stages of regeneration (Figure 4B, left). Additionally, several genes encoding ECM-modifying enzymes, such as matrix metalloproteases (MMPs) and Adam-proteases, were differentially regulated during regeneration (Figure 4B, right). Therefore, cardiac injury involves the upregulation of ECM components and their modifiers, potentially affecting mechanical properties in the heart.

To identify molecules corresponding with the stiffest compartment at the injury site (the DAPI^−^ region), we immunolabeled injured hearts at different stages of regeneration using antibodies against fibronectin and TenC. At 3 dpci, both molecules were present at the outer injury border (Figures S3A and S3B), with only slight fibronectin depositions detected at the center of the injury. At later regenerative stages (7, 14 dpci), TenC and fibronectin depositions were present at the outer injury border (Figure 4C), with fibronectin also surrounding the DAPI^−^ region (Figure 4C; Figure S3C, 21 dpci).

Previous studies reported that scarring in the injured zebrafish heart at 7 dpci involves collagen deposition at the injury center and a fibrin layer along the wound boundary^30^ (Figure S3D). Fibrin is a fibrous, insoluble protein produced at wound sites. During blood clotting, platelets bind thrombin molecules, converting fibrinogen into fibrin, forming a mesh around blood cells.^34^ At 3 dpci, acid fuchsin orange staining (AFOG) revealed high levels of fibrin at the injury site (Figure S3E), which became more restricted at 7 dpci (Figures S3D and S3F). Fibrin quantifications on heart sections (Figures S3E–S3H) showed that deposits became thinner from 3 to 7 dpci, then remained similar until 14 dpci (Figure S3I). However, longitudinal expansion only tended to decrease gradually from 3 to 14 dpci (Figure S3J). To investigate whether this layer coincided with the stiff DAPI^−^-region, we imaged heart sections stained with DAPI and phalloidin before AFOG counterstaining. Indeed, DAPI^−^-regions overlapped with fibrin deposits at 7 and 14 dpci (Figure 4D).

Next, we conducted environmental scanning electron microscopy (ESEM) of 100 μm heart sections to characterize fibrin deposits at 7 and 14 dpci and subsequently performed fluorescence labeling with DAPI and rhodamine phalloidin on ESEM-imaged heart sections (Figure 4E). At 7 dpci, the injury site was distinguishable from the healthy trabecular myocardium (Figure 4F). Moreover, ESEM images revealed that fibrin was organized as a scaffold and dense deposit near the wound border, whereas individual fibers were not apparent (Figure 4F). This organization was even more pronounced at 14 dpci, where the DAPI^−^-region exhibited a sponge-like morphology (Figure 4G, regions 1, 2), clearly distinguishable from the regenerating myocardium (Figure 4G, region 3). ESEM did not reveal the presence of cells within the fibrin layer, consistent with a reduced content of DAPI^+^ nuclei in this region (Figures 4F and 4G). In summary, our results revealed that a fibrin scaffold is the stiffest compartment at the injury site of the regenerating zebrafish heart.

### Genes involved in fibrin deposition and lysis are activated in the injured heart

**Figure 5:**
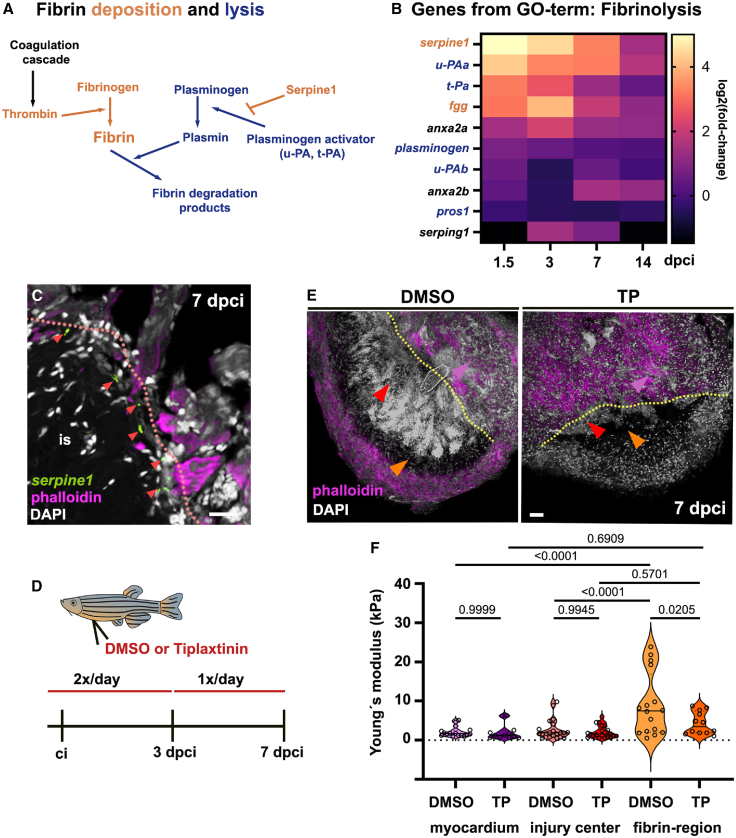
Serpine1-inhibition affects the stiffness of the fibrin scaffold (A) Scheme indicating factors involved in fibrin deposition (orange) and fibrin lysis (blue). (B) Heatmap showing RNA-sequencing results from heart apexes (see Figure 4A). Indicated are expression levels (log2 [fold-change]) of genes related to the GO-term “Fibrinolysis” at different stages of regeneration compared to non-injured heart tissue. (C) Representative image of fluorescence *in situ* hybridization on heart sections co-stained with phalloidin (magenta) and DAPI (white), indicating *serpine1*-expressing cells at the injury site (red arrowheads). The dotted line demarcates the injury site (is). (D) Scheme indicating experimental approach: Tiplaxtinin (TP) or DMSO was intraperitoneally injected into fish two times per day from after the cryoinjury until day 3 and once per day until 7 dpci. (E) Confocal images of DAPI (white) and phalloidin (magenta) stained heart sections after AFM-microscopy. Arrows indicate the injury center (orange), adjacent myocardium (purple), and the DAPI^−^ -/fibrin region (red). Dotted lines demarcate the injury site. (F) Violin plot indicating individual and median (black line) Young's modulus values measured with AFM on injured heart sections after DMSO or tiplaxtinin (TP) treatment. Ordinary one-way ANOVA with Tukey’s multiple comparison test results are indicated. Scale bars, 20 μm in C and E. See also Figure S4.

To investigate the molecules involved in the formation of the fibrin scaffold (Figure 5A), we specifically evaluated the bulk sequencing data for GO terms related to “fibrin clot formation” (GO:0072378; Figure S4A) and “fibrinolysis” (GO:0042730; Figure 5B). Fibrin is the principal component of blood clots that becomes activated upon tissue injury, initiating a coagulation cascade involving platelets, red blood cells, and endothelial cells, ultimately forming a blood clot to prevent further bleeding.^61^ During this process, the coagulation factor thrombin converts fibrinogen molecules into fibrin fibers, forming the core structure of the blood clot. Blood clot lysis involves plasmin activators converting plasminogen into plasmin, which degrades fibrin fibers. A key molecule controlling this degradation process is Plasminogen activator inhibitor 1 (PAI-1, referred to here as Serpine1), which blocks plasmin activators (Figure 5A).^62^ Our comparative whole-transcriptome analysis revealed that several genes encoding coagulation factors (e.g., f5, f3a, f2, and f1a1b) are strongly upregulated from early stages of regeneration (1.5–3 dpci) until at least 7 dpci (Figure S4A). Similarly, genes expressed by platelets, such as *filamin a* (*flna*), *integrin beta 3a* (*itgb3a*), *glycoprotein Ib platelet subunit beta* (*gb1bb*), and *platelet glycoprotein 9* (*gp9*), as well as other genes encoding factors involved in blood coagulation, such as *protein disulfide isomerase A4* (*pdia4*) and *hepsin* (*hpn*), were upregulated following cryoinjury of the zebrafish heart (Figure S4A).

The dataset from injured hearts also included several genes related to the GO term “Fibrinolysis.” These included genes related to fibrin deposition (highlighted in orange in Figures 5A and 5B), such as *serpine1* and *fibrinogen gamma chain* (*fgg*). Additionally, genes encoding proteins that promote fibrin clot lysis (highlighted in blue in Figures 5A and 5B) were differentially expressed in the injured heart, including *plasminogen activator, urokinase a* and *b* (*u-PAa*, *u-PAb*) and *plasminogen activator, tissue-type* (*t-PA*). In summary, the gene expression data analyses indicated that cryoinjury in the zebrafish heart triggers processes with molecular characteristics of fibrin clot formation and lysis, which impact fibrin deposition at the injury site.

### Serpine1 modifies tissue stiffness of the fibrin scaffold within the injured zebrafish heart

Fibrin deposition defined the stiffest compartment at the injury site during the later stages of cardiac regeneration. To further investigate how clot-forming mechanisms affect fibrin stiffness, we examined the role of Serpine1, a modulator of fibrinolysis (Figure 5A). Previous studies have reported that *Serpine1/serpine1* is upregulated following cardiac injury in both mice and zebrafish.^58^^,^^63^^,^^64^ Consistent with these findings, we detected *serpine1*-expressing cells at the injury site and near the DAPI^−^ region of the cryoinjured zebrafish heart at 7 dpci (Figure 5C). To functionally assess the role of Serpine1, we inhibited its activity through daily intraperitoneal injections of the inhibitor tiplaxtinin (TP)^58^ for six consecutive days post-cryoinjury (Figure 5D). We then measured tissue stiffness of the injury center, the fibrin region (DAPI^−^) and the injury-adjacent myocardium (Figures 5E and 5F) at 7 dpci on heart sections as described earlier (Figure 3A). In DMSO-injected control zebrafish, the center of injury exhibited mechanical properties similar to the myocardium adjacent to the injury site (Figure 5F; 2.7 ± 2.6 kPa and 2.1 ± 1 kPa, respectively). In contrast, the DAPI^−^/fibrin-positive region was much stiffer (Figure 5F; 9.0 ± 8 kPa), similar to our observations of hearts at 14 dpci (Figure 3J).

We next analyzed tissue stiffness following Serpine1 inhibition with TP, which did not affect the myocardium adjacent to the injury site (Figure 5F; 2.1 ± 1 kPa) or the center of the cryoinjury (Figure 5F; 2.1 ± 2 kPa). However, the inhibition of Serpine1 markedly affected the DAPI^−^/fibrin-positive region, which was notably softer in TP-treated samples (Figure 5F; 4.3 ± 3 kPa) compared to control hearts. Additionally, the significant stiffness differences between the DAPI^−^/fibrin-positive region and the adjacent myocardium and center of injury regions were eliminated in TP-treated samples (Figure 5F). These findings demonstrate that the loss of Serpine1, a component of the fibrin-regulating cascade (Figure 5A), promotes fibrinolysis, impacting tissue stiffness at the cryoinjury site in the regenerating zebrafish heart.

### Stiffness of the fibrin scaffold enhances regenerative Yap1 activity within endocardial cells at the injury site

**Figure 6:**
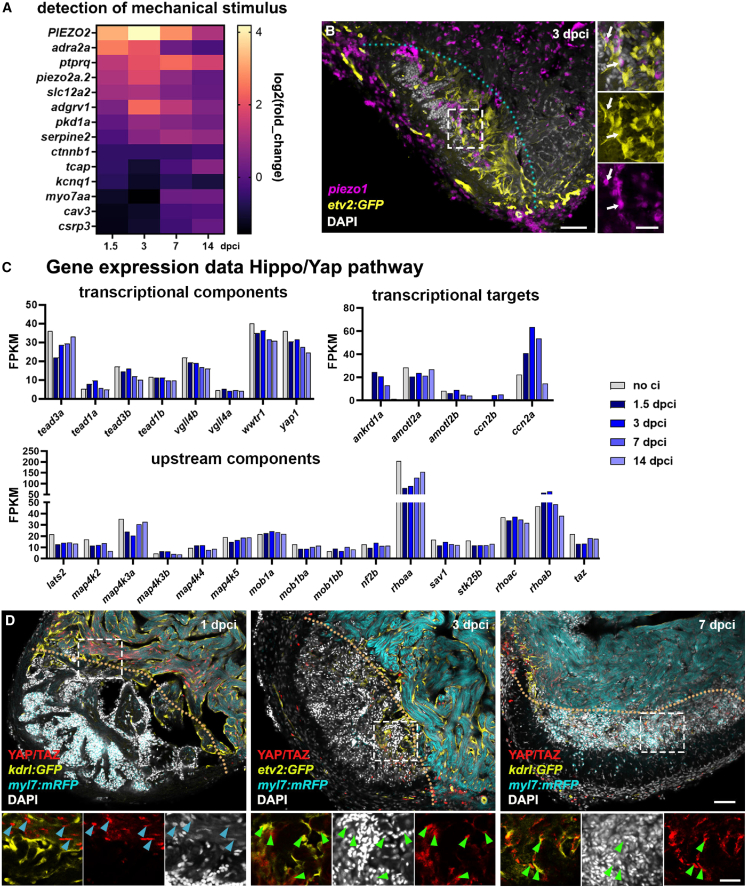
Biomechanical signaling in the regenerating zebrafish heart (A) Heatmap showing RNA-sequencing results from heart apexes (see Figure 4A). Indicated are expression levels (log2 [fold-change]) of genes related to the GO-term “Detection of mechanical stimulus” at different stages of regeneration compared to non-injured heart tissue. (B) Representative confocal image of fluorescent *in situ* hybridization for *piezo1* (magenta) on a heart section of *Tg(etv2:GFP)* endocardial (yellow) reporter fish. White arrows indicate *piezo1*^*+*^ endocardial cells at the injury site. (C) Bar charts showing RNA-sequencing results from non-injured (no ci) and injured apexes (1.5–14 dpci, see Figure 4A). Shown are FPKM (Fragments Per Kilobase of transcript per Million mapped reads) of genes encoding upstream and transcriptional components, and transcriptional targets of the Hippo/Yap pathway. (D) Confocal images of heart sections of endocardial reporter fish [*Tg(kdrl:EGFP)*^*s843*^*or Tg(etv2:GFP)*^*zf372*^] and *Tg(myl7:mRFP)*^*md3*^*,* immune-stained for YAP/TAZ (red). Arrows indicate nuclear localization of YAP in myocardium (blue) and endocardium (green). Dotted lines demarcate the injury site. Scale bars, 50 μm in B, D upper panels. 25 μm in B, D in lower panels. See also Figure S5.

Changes to biomechanical tissue properties can be transduced into biochemical signaling with consequences for cell proliferation, differentiation, polarity, and migration.^2^^,^^5^ To identify potential molecules that detect and transduce biomechanical stimuli in the regenerating zebrafish heart, we searched our gene expression datasets (Figure 4A) for genes related to the GO-Term “detection of mechanical stimulus” (GO:0050982) (Figure 6A) and “cellular response to mechanical stimulus” (GO:0071260) (Figure S5A). Genes encoding family members of the piezo-type mechanosensitive ion channel PIEZO2 and Piezo2a2 were differentially expressed in the RNAseq dataset (Figure 6A).

Piezo channels transduce the sensation of tissue stiffness into biochemical signaling.^16^^,^^17^ Whole-mount *in situ* hybridizations demonstrated the expression of *piezo1* and *piezo2a* at the injury site at 3 and 7 dpci (Figures S5B and S5C). Additionally, fluorescence *in situ* hybridization on heart sections of endocardial reporter fish, Tg(*etv2:GFP*), located *piezo1* expression in endocardial cells and non-endocardial cells at the injury site (Figure 6B), suggesting a potential role for mechanotransduction in the injured heart. The Hippo pathway is a key signaling pathway involved in forwarding biomechanical stimuli,^24^ and augmented nuclear YAP induces a proliferative program during cardiac regeneration in mammals.^27^^,^^28^ Therefore, we searched whole-transcriptome expression datasets for genes encoding Hippo pathway proteins (Figure 4A). This analysis revealed that several upstream Hippo components, Yap1, and Tead family members were expressed in the cryoinjured heart, with transcriptional target genes specifically upregulated at 1.5, 3, and 7 dpci (Figure 6C). To complement these results, we performed immunolabeling on tissue sections of injured hearts at 1, 3, and 7 dpci using an antibody against Yap1, which was localized to the nucleus, indicating the activation of Hippo pathway target gene expression. High-resolution confocal microscopy images at 1, 3, and 7 dpci showed nuclear localization of Yap1 within cardiomyocytes adjacent to the injury site (Figure 6D, blue arrows). Furthermore, at 3 and 7 dpci, nuclear Yap1 was observed in *kdrl*:GFP or *etv2*:GFP-expressing endocardial cells within the injury site (Figure 6D, green arrows), and in cells at the injury border, potentially epicardial cells or fibroblasts (Figure 6D).

**Figure 7:**
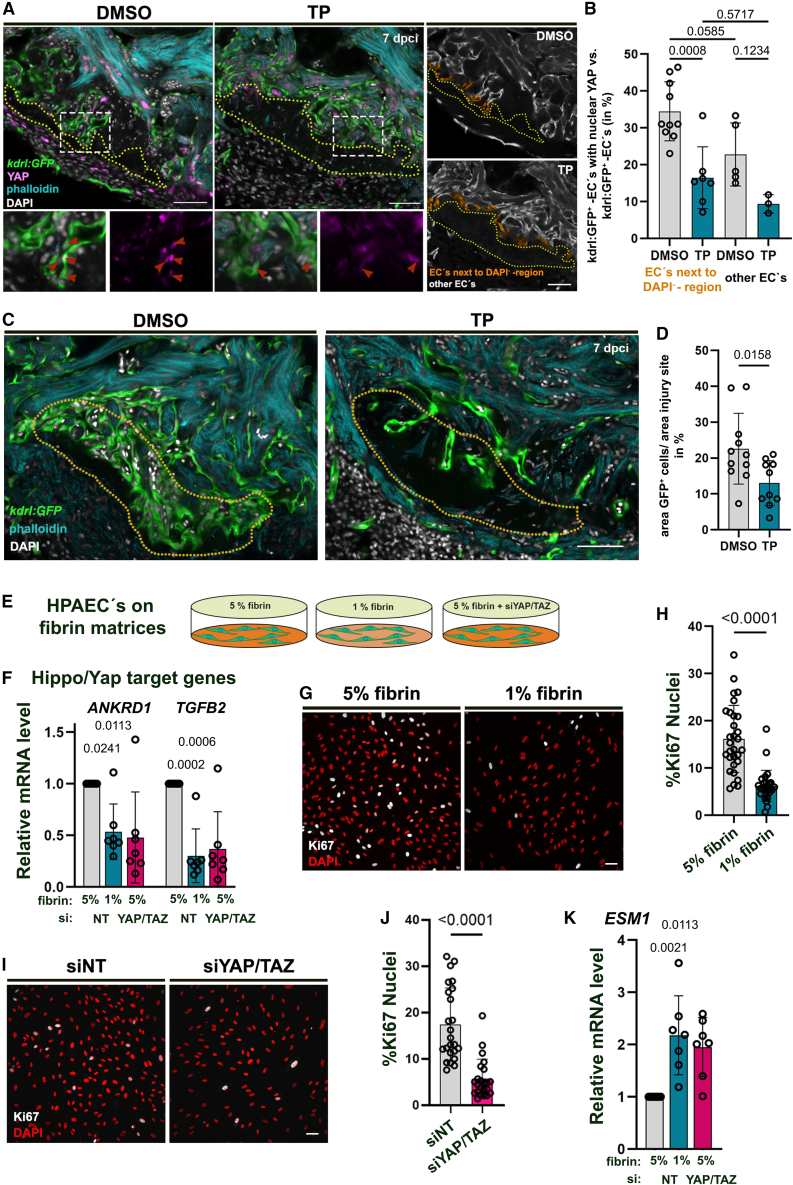
Fibrin stiffness affects YAP localization and cell proliferation (A) Confocal images of heart sections of endocardial reporter fish (*Tg[kdrl:EGFP]*^*s843*^) treated with DMSO or tiplaxtinin (TP) and immune-stained for YAP/TAZ (magenta), phalloidin (myocardium, turquois) and DAPI. Dotted lines demarcate the DAPI^−^ -region. On the left panel, endocardial cells adjacent to the DAPI^−^ -regions are labeled orange. (B) Bar chart indicating the percentage of endocardial cells next to the DAPI^−^ -regions or in the periphery of the injury with YAP-nuclear localization. Individual values and means (bars) +/− SD are represented.One-way ANOVA with Tukey’s multiple comparison test results are indicated. (C) Confocal images of heart sections of endocardial reporter fish *Tg(kdrl:EGFP)*^*s843*^ at 7dpci treated with DMSO or tiplaxtinin (TP). The dotted lines demarcate the inner injury site, where the GFP^+^ endocardium was quantified. (D) Bar chart showing the GFP-fluorescent signal at the inner injury site in DMSO or TP-treated hearts. Individual values and means (bars) +/− SD are represented.One-way ANOVA with Tukey’s multiple comparison test results are indicated. (E) Scheme indicating experiments with Human Pulmonary Arterial Endothelial Cells (HPAECs). (F) Bar chart indicating realative mRNA-levels of *ANKRD1* and *TGFB2* from HPAECs grown on 1% or 5% fibrin with siNT (nontargeting siRNA) or siYAP/TAZ. Individual values and means (bars) +/− SD are represented. One-way ANOVA with Tukey’s multiple comparison test results are indicated. (G) Representative images of HPAECs immune-stained for Ki67 and DAPI. (H) Bar chart showing the percentage of Ki67^+^ nuclei of HPAECs grown on 5% or 1% fibrin. Individual values and means (bars) +/− SD are represented. One-way ANOVA with Tukey’s multiple comparison test results are indicated. (I) Representative images of HPAECs immune-stained for Ki67 and DAPI. (J) Bar chart showing the percentage of Ki67^+^ nuclei of HPAECs grown with siNT or siYAP/TAZ. Individual values and means (bars) +/− SD are represented.*t* test results are indicated. (K) Bar chart indicating realative mRNA-levels of *ESM1* from HPAECs grown on 1% or 5% fibrin with siNT or siYAP/TAZ. Individual values and means (bars) +/− SD are represented.*t* test results are indicated. Scale bars, 50 μm in A upper panels, C, G, I. 25 μm in A in lower panels. See also Figure S6.

Immune-staining revealed that a portion of *kdrl:*GFP^+^ endocardial cells at the injury site that are contacting the DAPI^−^/fibrin-positive region, occasionally had a nuclear YAP/TAZ localization (Figure S5D). The functional endocardium is a crucial component for heart regenerative processes, regulating cardiomyocyte proliferation, the immune response, or as a source for cardiac fibroblasts.^39^^,^^65^^,^^66^ This prompted us to investigate whether the stiffness of the fibrin scaffold influences Hippo signaling activation in endocardial cells within the injury area. Using the established protocol, we treated zebrafish post-cryoinjury with the Serpine1 inhibitor TP (Figure 5D) and analyzed Yap1 localization specifically in endocardial cells adjacent to the DAPI^−^/fibrin-positive region (Figure 7A, highlighted in orange in right panels). In DMSO-treated control hearts, which exhibited higher tissue stiffness (Figure 5F), Yap1 localized to the nucleus in 34.5% (±8%) of *kdrl:*GFP^+^ endocardial cells adjacent to the DAPI^−^/fibrin-positive region (Figures 7A and 7B). In contrast, TP treatment reduced the percentage of endocardial cells with nuclear Yap1 in this region to 16% (±8%). Other *kdrl*:GFP^+^ endocardial cells at the injury site exhibited a slightly lower percentage of nuclear Yap1 (22.8% ± 9%), which was not significantly affected by TP treatment (9.4% ± 3%) (Figures 7A and 7B). These findings demonstrate that softening the fibrin scaffold through Serpine1 inhibition impacts the signaling of the Hippo pathway, a crucial regulatory pathway in cardiac regeneration, within adjacent endocardial cells.

To examine whether tissue softening affected endocardial regeneration, we analyzed *kdrl:*GFP^+^ endocardial expansion at the injury site. Indeed, fish treated with TP had less endocardium at the injury site compared to DMSO-treated fish (Figures 7C and 7D). This suggested that tissue softening-induced Hippo signaling negatively affected endocardial regeneration. We next investigated whether this correlation is conserved in human endothelial cells. Culturing human pulmonary artery endothelial cells (HPAECs) on matrices with different stiffnesses (1% fibrin, 100 Pa versus 5% fibrin, 500 Pa; Figure 7E) revealed that the softer matrix (1% fibrin) activated Hippo signaling as indicated by a lowered expression of the Yap target genes *TGFB2* and *ANKRD1* (Figure 7F). In line with this finding, the siRNA-mediated inhibition of YAP and TAZ in HPAECs cultured on a stiff matrix (5% fibrin) or on matrices with low (1% fibrin) (Figure 7F) or without fibrin also reduced the expression of *TGFB2* and *ANKD1* (Figure S6). To assess the role of Hippo signaling in endothelial cell proliferation, we quantified Ki67 in HPAECs grown on 1% and 5% fibrin and upon silencing of YAP/TAZ (Figures 7G–7J). Both reducing the tissue stiffness and inhibiting YAP/TAZ reduced cell proliferation (Figures 7G–7J). We also found that the expression of *ESM1*, a marker of tip cell identity and endothelial dysfunction,^67^ was upregulated in both conditions (Figure 7K). These results suggest that endothelial cells in contact with a stiffer matrix increase the translocation of Yap into the nucleus, triggering regenerative proliferation.

## Discussion

Our study provides the first detailed spatial and temporal characterization of the mechanical properties of the injured adult zebrafish heart. We established a correlation between these properties and biomechanical Hippo signaling, which regulates regenerative processes. Through fine-mapping tissue stiffness at the injury site, we identified distinct regions with characteristic mechanical properties. Notably, we found that fibrin deposits at the injury site contribute much more to tissue stiffness than collagen, with the injury center being much softer than the fibrin-rich periphery. We also discovered that the pharmacological inhibition of Serpine1, an inhibitor of fibrinolysis, critically alters the mechanical properties of the injury site, including the fibrin scaffold. These changes in stiffness of this compartment impact Hippo pathway biomechanical signaling, as evidenced by decreased levels of nuclear Yap1 in endocardial cells.

Upon cryoinjury, the zebrafish heart deposits fibrotic tissue rich in ECM. Such ECM deposits can dramatically alter the mechanical properties of the heart.^5^ Therefore, precise and high-resolution methods were required for assessing the location-specific mechanical properties of the regenerating heart. Different methods have previously been applied to measure mechanical properties in the zebrafish heart, yielding conflicting results. Garcia-Puig et al. used AFM to measure the ECM of a decellularized heart and observed decreased stiffness after cardiac injury.^68^ Conversely, a study employing micropipette aspiration to estimate ventricular stiffness reported an increase at 3 dpci, with a return to pre-injury stiffness between 14 and 35 dpci.^13^ However, the absolute stiffness values reported differed by 1–2 orders of magnitude from our results. In our study, we utilized a large-displacement depth-sensing nanoindenter approach to measure from outside the entire regenerating ventricle. This method revealed stiffness variations ranging from 4 to 11 kPa, but it did not show significant differences in ventricular compliance between injured and non-injured hearts. While a more detailed focal measurement using this approach would be preferable, the values clearly indicate that stiffness levels do not increase to those observed in injured mammalian hearts. In mammalian models, an AFM-nanoindentation approach showed a 3-fold stiffness increase from 18 ± 2 kPa up to 55 ± 15 kPa in infarcted ventricles following left coronary ligation (LAD).^4^ Therefore, comparing stiffness values obtained with different measurement methods must be critically reviewed, and mechanical property changes are different in zebrafish compared to the mammalian heart.

**Figure 8:**
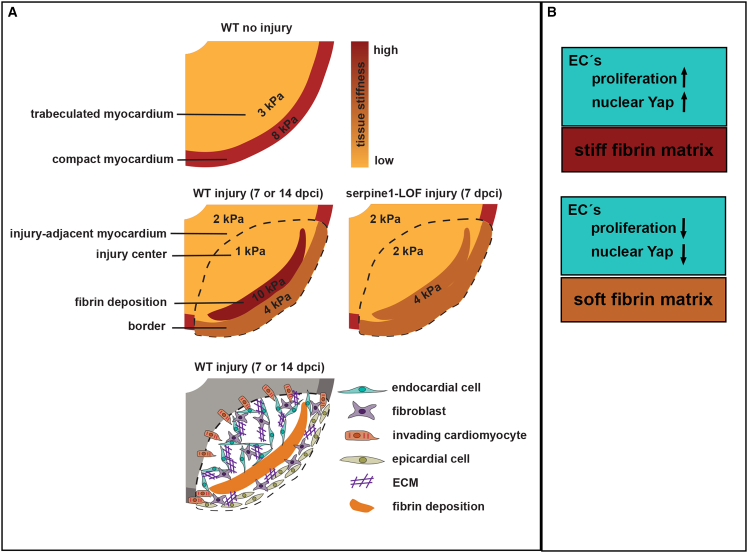
Model indicating stiffness measurement results (A) The upper and center panel approximate Young's modulus values of the myocardium and injury site. In the lower panel typical cell types and injury site components are shown for corresponding regions. In the non-injured heart the compact myocardium is stiffer than the trabeculated myocardium. Upon injury, the stiffest region corresponds with the fibrin deposition. Injury center and trabeculated myocardium have similar Young's modulus values. Upon Serpine1-inhibition, the stiffness of the fibrin-region is reduced compared to control hearts. (B) Scheme indicating the effects on endothelial/endocardial cells by a stiff or softer matrix.

In our study, the absolute Young’s moduli were found to be of the same order of magnitude when using large-displacement depth-sensing and colloidal probe-based AFM nanoindentation methods. Detailed measurements of Young’s modulus in specific regions of heart sections at various regeneration timepoints, conducted using AFM, identified a compartment with increased stiffness corresponding to the presence of a fibrin scaffold (Figure 8). Interestingly, this stiffer, fibrin-rich region exhibited mechanical properties similar to the compact myocardium, differing mechanically from the softer trabeculated myocardium (Figure 8). This suggested that fibrin deposits form a rigid structure at the wound site, maintaining the physical properties of the ventricle, resisting the mechanical load from cardiac pumping, while preventing ventricular rupture.^3^ Our initial nanoindentation measurements from the outside of the ventricle did not reveal significant changes in overall ventricular mechanical properties, likely due to the summation of the properties of tissue layers stacked on top of each other. Interestingly, the center of the injury, despite its high ECM content,^35^ is mechanically softer. This region is highly dynamic and contains regenerating endocardium, fibroblasts, and wound-invading cardiomyocytes. A softer environment may be more permissive and facilitate the proliferation and migration of these cells, consistent with previous studies. For instance, in a 3D culture, preosteoblasts embedded in soft gels exhibited increased motility and migrated longer distances compared to those in stiffer gels.^69^ Also, cardiac fibroblasts are influenced by the mechanical conditions of their environment, with soft substrates (10 kPa) inhibiting expansion, activation, and collagen production when compared to stiffer substrates (40 kPa).^70^ Similarly, cardiomyocytes respond to environmental physical properties regarding their function, differentiation state, and proliferation.^15^ IPS-derived cardiomyocytes cultured on soft (12 kPa) or medium-soft (30 kPa) hydrogels demonstrated improved contractile function and lower expression of ECM components compared to those on stiff substrates (123 kPa).^71^ This suggests that in the regenerating zebrafish heart, a stiff outer mechanical shield provides structural support, while a softer core facilitates cell proliferation and migration (see model, Figure 8). Future studies will need to explore whether perturbations in tissue stiffness within these regions can affect regeneration processes. A previous study suggested a potential correlation between increased ECM production and cardiomyocyte migration, both of which are impaired in *il11ra* mutants.^65^ Further investigation is needed to determine if mechanical properties are altered in this mutant context.

Here, we demonstrate that the fibrin scaffold constitutes the stiffest compartment in the injured zebrafish heart during the reparative phase (Figure 8A). In mammals, fibrin deposits have been studied only marginally. For instance, during viral myocarditis, interstitial fibrin deposits are apparent at 14 and 30 days post-infection.^72^ Following myocardial infarction, the plasma-derived provisional matrix includes fibrin fibers and serves as a scaffold for immune cells, endothelial cells, and fibroblasts during the early inflammatory phase, similar to the zebrafish heart.^30^^,^^73^ During subsequent proliferative and reparative stages, the fibrin network is lysed and replaced by fibronectin and collagens.^73^ This process contrasts with our observations in zebrafish, where a fibrin cap persists until the late stages of regeneration, which is merely surrounded by fibronectin (14 dpci, Figure S3G).

The increase in cardiac stiffness in the diseased mammalian heart has been attributed to intracellular factors, such as the giant molecule TITIN in cardiomyocytes,^74^ and extracellular factors, including ECM molecules secreted by activated fibroblasts.^2^ Myocardial infarction-induced fibrosis leads to increased ventricular stiffness in rat hearts,^4^ along with heightened collagen accumulation and cross-linking, which can determine the rigidity of fibrotic cardiac tissue.^59^ In our study, we noted a tendency toward greater rigidity in regions rich in Col1a1. This region also contains both cardiomyocytes and fibroblasts^55^ and shares characteristics of diffuse myocardial fibrosis found in the diseased mammalian heart. However, our findings show that collagen is not the main contributor to increased tissue stiffness in the injured zebrafish heart and does not perturb mechanical properties to the same extent as in mammalian hearts.^75^

Although fibrin deposits have been previously noted,^30^ the deposition and resolution of a fibrin network in the zebrafish injured heart has never been studied. During tissue wounding, blood plasma-derived fibrinogen forms a mesh of fibrin fibers, providing structural and mechanical support to blood clots.^34^ Therefore, controlling the coagulation and lysis of clots is crucial to prevent severe blood loss and maintain blood flow. The molecular cascade of regulatory coagulation proteins is conserved between mammals and fish,^43^ with the zebrafish embryo serving as a well-established model in hemostasis and thrombosis research.^44^ In this study, we report the expression of several components of the fibrin deposition and lysis cascade during cardiac injury and subsequent regeneration. It has been shown that the rigidity of fibrin deposits is modulated by the presence of thrombin and fibrinogen, which both promote fibrin deposition (Figure 5A^76^), as well as by the crosslinking of fibrin fibers.^77^^,^^78^

We identified a role for Serpine1, an inhibitor of urokinase plasminogen activator (uPA) and tissue plasminogen activator (tPA), in determining the stiffness of fibrin deposits. When Serpine1 levels are high, plasminogen cannot be converted to plasmin, preventing fibrin degradation (Figure 5A). We suggest that reducing Serpine1 increases the efficacy of plasminogenactivators, promoting the conversion of plasminogen to plasmin and inducing fibrin degradation (Figure 5A). An increased density of fibrin clots has been correlated with increased stiffness.^79^ Previous studies in fish showed that a short-time inhibition of Serpine1 for the first 10 days does not affect heart regeneration at long term when examined at 22dpci,^58^ suggesting that the heart can recover from a transient fibrin softening. However, the majority of studies have focused on fibrin networks *in vitro* or within artificially generated fibrin gels. Understanding the mechanical properties of fibrin deposits and their modulation holds significant importance for thrombosis research. Our study demonstrates the suitability of the adult zebrafish heart as an effective model for investigating fibrinolytic processes following tissue injury *in vivo*.

We report that Yap1 has a nuclear localization in the endocardium of the injured zebrafish heart. This biomechanical signaling process is influenced by Serpine1 inhibition, which induces alterations in tissue stiffness. It is well-established that mechanical cues activate Hippo pathway signaling in endothelial cells, including factors such as physical pressure, strain, cell geometry, and the stiffness of the extracellular environment.^24^^,^^80^ The transcriptional regulators YAP/TAZ regulate endothelial cell proliferation, migration, and survival.^80^ In the zebrafish heart, the endocardium provides crucial signals for regulating myocardial proliferation, fibrotic tissue deposition,^65^ and inflammatory processes.^65^^,^^66^^,^^81^ Previous reports have shown that the Yap1 target gene *cnn2a*/*cbfga* is expressed in endocardial cells, where it regulates ECM production and myocardial regeneration.^82^ However, little is known about the processes that regulate the regeneration of the endocardium itself. The injury endocardium is highly dynamic, evolving from a disorganized mass at 3 dpci into a more structured endocardial organization within the injury site by 9 dpci.^58^ Here, we show that a softer fibrin matrix was accompanied by a reduction in endocardial expansion at the injury site. During zebrafish cardiac chamber growth, increased nuclear Yap1 correlates with heightened endocardial proliferation, both triggered by augmented tensile forces on endocardial cell junctions due to an expansion of myocardial chamber dimensions.^83^ In line with this, our experiments on HPAECs showed that cell proliferation depends on the stiffness-induced nuclear translocation of YAP (Figure 8B). We propose that fibrin stiffness in the injured heart serves as a crucial mechanical cue that influences Yap1 nuclear localization, potentially inducing endocardial proliferation and tissue expansion at the injury site. The mechanisms by which environmental mechanical properties are sensed and transduced to the Hippo pathway remain to be elucidated.

We have observed the expression of genes encoding Piezo channels in the injury endocardium, suggesting their involvement in regenerative processes. Biomechanically stimulated Piezo channels indeed regulate Yap nuclear localization in various tissues, including during outflow tract morphogenesis in the zebrafish heart and in human aortic valves.^84^^,^^85^ Our findings uncover distinct biomechanical pathways in the regenerating heart and introduce a pharmacological tool to modulate biomechanical properties and signaling. This advancement significantly contributes to future studies on the role of biomechanics in heart regenerative processes.

In summary, our study introduces a powerful novel method for measuring tissue stiffness in the zebrafish heart, yielding highly detailed insights into the different regions of injured cardiac tissue. The resulting stiffness maps reveal a fine grid of areas with varying stiffness, which is crucial for understanding mechanotransduction and signaling within regenerating zebrafish hearts. We have identified fibrin stiffness as a critical determinant in regulating biomechanical signaling, which is a prerequisite for successful cardiac regenerative processes.

### Limitations of the study

There are limitations to our study. We identified different regions of stiffness in the injured zebrafish heart. Our study focused on the stiffest compartment, the fibrin scaffold, which was most prominent from 7 dpci. Future studies should also investigate the stiffness changes early upon injury. A more detailed analysis with higher resolution of AFM measurements would be necessary to detect regions of different stiffness. Also, we show that Serpine1-inhibition leads to the softening of the fibrin deposit and changes in Hippo/YAP signaling by using TP. It remains to be tested whether other molecules from the fibrinolysis cascade similarly affect fibrin stiffness and biomechanical signaling. In a future approach, zebrafish mutants for those molecules should be tested for Hippo/Yap activity, and finally, this could help to test if modifying the fibrinolysis cascade affects regeneration in the long term.

## Resource availability

### Lead contact

Requests for further information and resources should be directed to and will be fulfilled by the lead contact, Salim Abdelilah-Seyfried (salim.seyfried@uni-potsdam.de).

### Materials availability

This study did not generate new unique reagents.

### Data and code availability

• Data: The raw and analyzed RNA sequencing data were deposited at Gene Expression Omnibus with the accession number: GEO: GSE297624 and are publicly available as of the date of publication.
• Code: This study did not generate a new code.
• Other: Any additional information required to reanalyze the data reported in this paper is available from the lead contact upon request.

## Acknowledgments

We would like to thank all members of the Seyfried group for their critical reading and comments on the article. Thanks to O. Baumann for his support of confocal microscopy and A. Kühnel, A. Hubig, S. Weiche, B. Wuntke, and R. Dünnebacke for technical support, and thanks to A. Kühnel, F. Viyof Ful and F. Sow for fish husbandry. S.A.-S. was generously supported by SFB958, 10.13039/501100001659Deutsche Forschungsgemeinschaft projects SE2016/7-3, SE2016/10-1, SE2016/13-1, SE2016/16-1, the Leducq Transatlantic Network of Excellence “21CVD03– ReVAMP,” the Marie Skłodowska-Curie Innovative Training Network (ITN) “V.A.Cure,” and a Kickbox Seed Grant and Flexible Funds 2017 from the 10.13039/501100015678Einstein Center for Regenerative Therapies, Charité Berlin. The LSM 880 AiryScan was funded by a DFG grant INST 336/114-1 FUGG.

## Author contributions

J.M.: conceptualization, resources, software, formal analysis, investigation, visualization, methodology, data curation, supervision, writing – original draft, writing – review and editing, and funding acquisition. T.P.: resources, formal analysis, investigation, visualization, and methodology. I.T.: methodology and data curation. L.S.: methodology. C.J.R.: methodology. SA: methodology and software. P.F.: funding acquisition and investigation. K.B.: conceptualization, funding acquisition, and supervision. O.C.: conceptualization, funding acquisition, and supervision. S.S.: conceptualization, resources, supervision, funding acquisition, investigation, writing – original draft, project administration, and writing – review and editing.

## Declaration of interests

The authors declare no competing interests.

## Declaration of generative AI and AI-assisted technologies in the writing process

During the preparation of this work, the author(s) used GPT.UP – an AI tool for the University of Potsdam - in order to check grammar and language. After using this tool/service, the author(s) reviewed and edited the content as needed and take full responsibility for the content of the publication.

## STAR★Methods

### Key resources table

**Table:** 

REAGENT or RESOURCES	SOURCE	IDENTIFIER
**Antibodies**		

anti-GFP	Aves labs	Cat# GFP-1020; RRID: AB_10000240
anti-myosin light chain kinase (MLCK)	Sigma- Aldrich	Cat# M7905; RRID: AB_477243
anti-YAP/TAZ	Cell Signaling Technology	Cat# D24E4
anti-fibronectin	Sigma	Cat# F3648; RRID: AB_476976
TenascinC	US Biological	Cat# T2550-23; RRID: AB_2271850
Rhodamine-phalloidin	Invitrogen	Cat# R415
Phalloidin – Alexa Fluor 647	Thermo Fisher	Cat# A22287
DAPI	Thermo Fisher	Cat# D1306
Fluorescein goat anti-chicken IgY	Aves labs	Cat# F-1005; RRID: AB_2312516
anti-Ki67	Abcam	Cat# ab16667; RRID: AB_302459
anti-TAZ	Sigma- Aldrich	Cat# HPA007415; RRID: AB_1080602
anti-YAP	Santa Cruz	Cat# 10111999
anti-Collagen1a1, SP1.D8	Developmental Studies	Hybridoma Bank; AB_528438
Donkey antiMouse IgG (H + L) Alexa Fluor™ 555	Invitrogen	Cat# A-31570
Donkey antiRabbit IgG (H + L) Alexa Fluor™ 647	Invitrogen	Cat# A31573
Anti-Digoxigenin-AP, Fab fragments	Roche	Cat# 11093274910; RRID: AB_514497
Anti-Digoxigenin-POD, Fab fragments	Roche	Cat# 11207733910; RRID: AB_514500

**Chemicals, peptides, and recombinant proteins**		

Tiplaxtinin (PAI-039)	Selleckchem	S7922
Corning® Cell-Tak(TM)	MERK	CLS354240
Fibrinogen (human)	Sigma-Aldrich	T6884
Lipofectamine RNAiMAX	Invitrogen	13778150
Endothelial Growth Medium (EGM-2)	Lonza	CC-3162

**Critical commercial assays**		

Quick-RNA Microprep Kit	Zymo Research	R1050
Tyramide SuperBoost™ Kit	Thermo Fisher	B40942
SlowFade Gold	Invitrogen	S36936
RNeasy Mini Kit	Qiagen	74104
Super-Script III First Strand Synthesis System	Invitrogen	18080051
SYBR Green Master Mix	Applied Biosystems	A25742

**Deposited data**		

Raw and analyzed data	This paper	GEO:GSE297624

**Experimental models: Organisms/strains**		

Zebrafish:Tg(myl7:mRFP)^md3^	Rohr et al. 2008^57^	ZFIN: ZDB-ALT-080917-1
Zebrafish: Tg(etv2:GFP)^zf372^	Veldman and Lin 2012^86^	ZFIN: ZDB-ALT-130404-2
Zebrafish: Tg(kdrl:EGFP)^s843^	Beis et al. 2005^87^	ZFIN: ZDB-ALT-050916-14
Zebraifsh: Tg(col1a2:LOXP-mcherry-NTR)^cn11^	Sánchez-Iranzo et al. 2018^36^	ZFIN: ZDB-ALT-170711-14

**Experimental models: Cell lines**		

Human Pulmonary Artery Endothelial Cells (HPAECs)	Lonza	CC-2530

**Oligonucleotides**		

ON-TARGETplus Human TAZ siRNA	Horizon Discovery	L-016083-00- 0010
ON-TARGETplus Human YAP1 siRNA Smart Pool siRNA	Dharmacon/Horizon Discovery	L-012200-00- 0010
ON-TARGETplus Non-targeting Control Pool 50 nM Dharmacon	Dharmacon/Horizon Discovery	D-001810-10
Primer:CYR61_Fwd CAGGACTGTGAAGATGCGG	This paper	N/A
Primer:CYR61_Rev GCCTGTAGAAGGGAAACGCT	This paper	N/A
Primer:CTGF_Fwd GTTTGGCCCAGACCCAACTA	This paper	N/A
Primer:CTGF_Rev GGCTCTGCTTCTCTAGCCTG	This paper	N/A
Primer:ANKRD1 Fwd AGCGCCCGAGATAAGTTGCT	This paper	N/A
Primer:ANKRD1_Rev GTCTTTGGCGTTGAGGTCTGC	This paper	N/A
Primer:TGFB2_Fwd CCCCGGAGGTGATTTCCATC	This paper	N/A
Primer:TGFB2_Rev GCGGGATGGCATTTTCGGAG	This paper	N/A
Primer:NOTCH1_Fwd CGTAGATGACCTGGGCAAGT	This paper	N/A
Primer:NOTCH1_Rev TTAGCCCCGTTCTTCAGGAG	This paper	N/A
Primer:DLL4_Fwd TGACCACTTCGGCCACTATG	This paper	N/A
Primer:DlLL4_Rev AGTTGGAGCCGGTGAAGTTG	This paper	N/A
Primer:ESM1_Fwd GGGAGAAACTTGCTACCGC	This paper	N/A
Primer:ESM1_Rev CATGTCATGCTCTTTGCAG	This paper	N/A

**Software and algorithms**		

ImageJ		https://imagej.net/ij/
Adobe Photoshop 2021		https://www.adobe.com
GraphPad Prism9		https://www.graphpad.com

### Experimental model and study participant details

#### Zebrafish husbandry

Handling of zebrafish was done according to FELASA guidelines^88^ in compliance with German and Brandenburg state law, carefully monitored by the local authority for animal protection (LAVG, Brandenburg, Germany, Animal protocol #2347-21-2020). The following zebrafish lines were used: *Tg(myl7:mRPF)*^*md3*^,^57^ *Tg(etv2:GFP)*^*zf327*^,^86^ *Tg(kdrl:EGFP)*^*s843*^,^87^ *Tg(col1:mcherry-NTR)*.^36^ Cryoinjury and heart dissection were performed on male and female fish, 5 to 12 months of age.^56^

#### Zebrafish treatment

Adult zebrafish were anesthetized with 0.04% tricaine (Sigma, St Louis, MO, USA) and injected intraperitoneally with 30μl Tiplaxtinin (PAI-039, S7922, Selleckchem; 1500 μM) or DMSO in PBS.^58^ The first injection was done just before the cryoinjury. The treatment regimen is indicated in the corresponding figure.

#### Cell culture

Human Pulmonary Artery Endothelial Cells from Lonza, between passages 5 and 7, cultured in endothelial growth medium (EGM-2) containing 5% heat-inactivated fetal bovine serum (FBS) and endothelial cell growth supplements from Lonza at 37 °C and 5% CO_2,_ were used for the experiments. Fibrinogen (human) from Sigma-Aldrich T6884 was dissolved in sterile PBS at 10mg/mL and filter sterilized, and Thrombin stocks were prepared at 10 U/mL in 2mM Calcium chloride. 1% Fibrin (100 Pa) and 5% Fibrin (500 Pa) were prepared using 1mg/mL or 5mg/mL Fibrinogen and 0.5 U/mL or 1 U/mL thrombin, respectively, and allowed to polymerize for 1hour in the incubator. Cells were seeded at 10000/cm^2^ in 8-well chamber slides and cultured for 48 hours before being fixed for immunofluorescence or mRNA extraction for qPCR.

siRNA transfection was carried out using Lipofectamine (Invitrogen). mRNA extraction was done 48 hours post-transfection. ON-TARGETplus Human TAZ siRNA and ON-TARGETplus Human YAP1 siRNA Smart Pool siRNA from Horizon Discovery were used at 25nM to knock down TAZ and YAP, respectively.

### Method details

#### Tissue processing and staining procedures

Following dissection, hearts were washed in PBS and fixed overnight in 4% paraformaldehyde (PFA). After washing in PBST, hearts were embedded in 4% agarose/ PBS and sectioned at 50 μm using a LEICA vibratome. For immunostainings, heart sections were washed for 2 hours with PBST and Triton X-100 (0.5) and incubated for 20 minutes in 3% H_2_O_2_ before the blocking reaction (2 h) and antibody incubation. The following antibodies were used: GFP (1:500, Aves, chicken, GFP-1010), collagen type 1A1 (1: 20, DSHB, SP1.D8, mouse, C#SP1.D8, RRID: AB_528438), Tenascin-C (1:500, USBiological, rabbit T2550-23. MLCK (Sigma, St Louis, MO, USA), YAP/TAZ (1:100, Cell Signaling Technology, rabbit), fibronectin (1:100, Sigma, rabbit, F3648). Primary antibodies were detected using the Tyramide SuperBoost™ KitTyramide SuperBoost Kit (Thermo Fisher). For *in situ* hybridization (ISH) vibratome sections were dehydrated with methanol for storing and rehydrated through a methanol series. Sections were treated with 10μg/ml proteinase K for 40 min and refixed with paraformaldehyde (PFA). Sections were incubated with mRNA-probes (with digoxigenin) over night at 65°C. After several washes in phosphate-buffered saline with Tween20 (PBST) sections were incubated over night at 4°C with a probe-detecting antibody (Anti-digoxigenin, coupled to a alkaline phosphatase or a peroxidase, ROCHE).^89^ Finally, sections were stained in NBT/BCIP solution (ROCHE). For fluorescent ISH, we used the Tyramide SuperBoost™ KitTyramide SuperBoost Kit (Thermo Fisher). For imaging, heart sections were mounted in SlowFate (Invitrogen). Further stainings were done on paraffin sections. Hearts were dehydrated, embedded in paraffin, and sectioned at 7μm. For ISH paraffin sections were dehydrated, treated with proteinase K and refixed with PFA. Incubation with mRNA-probes was done over night at 65°C. After washes with maleic buffer, sections were incubated with a probe-detecting antibody coupled to a peroxidase (ROCHE).^90^ To reveal fluorescent signal we used the Tyramide SuperBoost™ KitTyramide SuperBoost Kit (Thermo Fisher). ISH was combined with immunostaining.^58^ Acid Fuchsin Orange staining (AFOG) was done on paraffin heart sections.^39^

HPAECs were fixed with 4% paraformaldehyde at room temperature for 10 minutes or methanol for 2 minutes on ice, followed by permeabilization with 0.2% TritonX in PBS for 10 minutes. CAS Block was used as blocking agent for 1 hr. Cells were incubated overnight with primary antibodies as indicated and secondary antibodies for 1 hr at 1:400 dilution. Rabbit anti-Ki67 (Abcam ab16667; 1:400), Rabbit-TAZ (Sigma- Aldrich HPA007415; 1:100), Mouse-YAP (Santa Cruz 10111999; 1:100), anti-rabbit 647, and anti-mouse 555.

#### Depth-sensing nanoindentation

Dissected hearts were washed with PBS with 0.1M KCl and fixed on a Polyspan-Cube with a dissection needle. Hearts were covered with PBST-KCl solution during measurements. Depth-sensing nanoindentation studies were performed using a Triboindenter TI950 (Hysitron, MN, USA) equipped with an extended stage (XZ-500) allowing maximum displacement of 500 μm. A standard transducer and diamond cono-spherical tip (r=100 μm) were used for the measurements. A displacement rate of ∼15 μm/s was used to approach, contact and load the sample. The tip was indented at various locations all over the heart. Data points shown in Figure 1D represent single measurements.

#### Atomic force microscopy (AFM) nanoindentation

For AFM-imaging dissected hearts were washed with PBS with 0.1M KCl. And subsequently embedded in 4% agarose for vibratome sectioning at 100μm. Sections were sticked on glass-bottom dishes coated with Corning™ Cell-Tak (Thermo Fisher Scientific) and PBS was added for measurements. The AFM-nanoindentation measurements were performed with a Nanowizard 3 (JPK instruments Inc.) equipped with a motorized precision stage (JPK instruments Inc.) coupled with a fluorescence microscope (Olympus IX83). The fluorescence microscope was equipped with a SOLA light engine® LED (lumencor®) and a water-cooled CMOS camera (Zyla 4.2P, Andor, Oxford Instruments). To shield the setup from vibrations, it was positioned on an active vibrator isolation stage (Accurion, Accurion GmbH) in a noise isolation box (JPK instruments Inc.). Heart tissue sections were probed using CP-Cont-PS-D cantilever with a 10.8 μm diameter polystyrene bead (nominal spring constant: 0.2 N/m, sQube, NanoAndMore GmbH). The cantilever sensitivity and spring constant was calibrated with PBST on a cover slip before the measurement using the contact mode and the build-in thermal noise method.^91^ The values for the spring constant are in the range of 0.15-0.3 N/m. The region of interest (ROI) was first examined with bright field and fluorescence microscopy using a 20x objective. Images of the ROI were taken for every region. Nanoindentation maps were acquired in the ROI using the QI-mode using 32x32 point grids with 20x20 μm size. The set point force was 16 nN and a z-length of 6 μm was used with a pixel time of 100 ms (speed: 150 μm/s). 2-4 nanoindentation maps were collected in 2-3 different regions on the same heart sample (see Figure 2F). Subsequently, after AFM measurements, heart sections were fixed in 4% PFA overnight. After washing with PBST, hearts were processed to immunolabelling and confocal imaging.

#### Environmental scanning electron microscopy (ESEM)

For ESEM, we used 50μm vibratome sections of previously fixed hearts. These were washed in water several times and mounted on a metal plate. The sample was precooled at 7°C. High-resolution imaging was done using a Quattro S ESE microscope (Thermo Fisher Scientific, FEI) at 3°C working temperature.

#### RNA extraction and quantitate ive PCR from HPAECs

RNA was extracted using RNeasy Mini Kit (Qiagen) from 200k to 300k cells per sample and 500 ng of RNA was used to synthesize cDNA using Super-Script III (Invitrogen). qPCR was performed in triplicates per sample with SYBR Green Master Mix (Applied Biosystems) using gene-specific primers and 1uL of cDNA per reaction.

#### RNA-sequencing

Hearts were dissected at 1.5, 3, 7,14 dpci and without injury and washed in ice-cold PBST. The apex of the ventricle was removed and 3-4 apexes in one tube were immediately frozen in liquid nitrogen and stored at -80°C. Four samples per timepoint were prepared. For RNA-extraction lysis buffer of Quick-RNA Microprep Kit (Zymo Research) was added and cells were lysed by sonication in a waterbath (BIORUPTOR Plus, Diagenode). We applied three cycles of sonication for 30 sec with 30 sec of pause in between. RNA was then extracted with Quick-RNA Microprep Kit (Zymo Research). The following steps (library preparation, sequencing and data analysis) were done by EXIQON. CDNA libraries of 4 samples per timepoint were prepared with Illumina TruSeq Stranded Total RNA Library Prep Kit and run on Illumina platform for sequencing. Reads were aligned to the *Danio rerio* reference genome (GRCz10, Ensemble_90) and mapped for quality control. On average 54.2 million reads were obtained for each sample and the average genome mapping rate was 66.5%. The number of identified genes per sample was calculated based on alignment to the reference genome. When performing statistical comparisons between groups, we included all genes irrespective of abundance. FPKM (Fragments Per Kilobase of transcript per Million mapped reads) values were normalized with the median of the geometric mean.^92^ To identify differentially expressed genes, it is assumed that the number of reads produced by each transcript is proportional to both its size and abundance. For analysis Tuxedo suite was used, including the Cufflinks, Cuffmerge and Cuffdiff steps. Differential expression analysis was done by averaging the value of the four samples for each time point and comparing samples of different time points post-injury with non-injured samples.

For identification of genes related to a specific Gene Ontology (GO-term), we compared published gene lists (https://amigo.geneontology.org/amigo) of specific terms (fibrinolysis, fibrin clot formation, cellular response to mechanical stimulus, detection of mechanical stimulus) with our gene lists obtained from differential gene expression analysis. Relative gene expression comparing no-injured and injured hearts (at 1.5, 3, 7, or 14 dpci) was represented in heat maps (Figures 4B, 5B, 6A, S5A, and S6A).

### Quantification and statistical analysis

#### Imaging and quantifications

Fluorescence imaging was done on ZEISS LSM710 and LSM 880 confocal microscopes. AFOG-stained heart sections and conventional ISH were imaged with an Axioskop (Zeiss) with an EOS 5 D Mark III (Canon) camera using a 10 or 20x objective. For image processing and quantifications, we used Fiji software^93^ and Adobe Photoshop. For a detailed analysis of DAPI^+^ content (Figure 3G), regions (center DAPI^+^, border DAPI^+^, in between DAPI^-^) were defined by eye on confocal images following DAPI^+^ nuclei density and location. Then DAPI^+^ nuclei were counted in small areas, whose size was measured. The number of DAPI^+^ nuclei per μm^2^ was then calculated. 3-4 sections, where the injury site was perfectly visible, were analyzed per heart, and 3-4 hearts per time point (7, 24, 21 dpci) were inspected. To compare the size of fibrin depositions at different timepoints (3, 7, 14, 21 dpci), we measured the length and the thickness of fibrin depositions (strong orange) on AFOG-stained heart sections (Figures S3E–S3I). Three measurements of the width were averaged to obtain one width value per section. At least 3 representative sections were analyzed per heart, and 2-4 hearts were analyzed per timepoint. For endocardium quantification, one confocal stack of immune-stained vibratome sections was used to measure the area of the inner injury site (Figure 7A), which was compared to the *kdrl:GFP*^*+*^- area within this region. One or two sections were analysed per heart with Fiji software.^93^ Proliferation counts of HPAECs were done by counting the DAPI positive nuclei per field of view (FOV) using the Analyze particle command in Fiji and by manually counting the Ki67 positive nuclei in the same FOV. On dot plotted represents the the relative number of Ki67 positive nuclei in one FOV. Each has 100 to 200 cells and 7 to 10 FOVs were taken per experiment.

#### Depth-sensing nanoindentation data analysis

Nanoindentation data were analysed using OriginPro and the Hertzian contact model in which tip radius, contact depth, and force were used to fit the initial contact (h<30 μm) and calculate elastic modulus.

#### AFM-data analysis

The acquired nanoindentation maps were analyzed using the JPK SPM data processing software (version spm-6.1.41, JPK Instruments Inc.). The approach curve was first baseline corrected and the height measured was corrected for cantilever bending. The contact point was determined (automatically adjust the x offset) and the slope and height of the approach curve were fitted to correct for offset (now the vertical tip position is fitted).

The built-in Hertz-Sneddon model^94^^,^^95^ was fitted to the approach/extend curve using a spherical tip shape (radius 5.4 μm) and a Poisson ratio of 0.5. Example data sets representing the force curves and the corresponding fit are shown in Figure S1. Frequency distributions in Figure 2B present stiffness values of several maps from either cortical or trabeculated myocardium from one heart. For comparisons, the median of the stiffness values of 2-4 indentation maps from one region was calculated (E_A_ in Figure 2F), followed by averaging those values of different regions within the injury site or from healthy regions (E_final_ in Figure 2F). One data point on the graph arose from one heart section. From stiffness measurements with region identification (Figures 3A–3C, 3H, 3J, 5E, and 5F) data were similarly analyzed specifically for values from indicated regions: myocardium, injury center, border in Figures 3B and 3C; myocardium, injury center (DAPI^++^), DAPI^−^, col1^+^ or MLCK^+^ in Figures 3H and 3J; myocardium, injury center (DAPI^++^) and fibrin region (DAPI^−^) in Figures 5E and 5F. Regions of the injury site were identified on confocal images of immune- and DAPI-stained sections (see above in “tissue processing and staining procedures”).

#### Statistical analysis

All statistical analyses were performed either with GraphPad Prism (version 6) or with Excel 2016 (Microsoft Office). For comparing several datasets, we used One-way ANOVA combined with Tukey’s multiple comparison test. *p*-values are indicated in the figures. All indicated *p*-values are two-tailed, and significance was defined as *p*-value <0.05. To compare the variation of AFM-stiffness measurement data, we further applied the f-test (Figure 3). Statistical tests are indicated in the figure legends.
