# Supplementary material for: Fibrin defines tissue stiffness and biomechanical signaling in regenerating zebrafish hearts as revealed by high-resolution stiffness mapping
Source: iScience. 2026 Mar 4;29(4):115231. doi: 10.1016/j.isci.2026.115231 (PMC13049610; doi:10.1016/j.isci.2026.115231)
Supplement: Document S1. Figures S1–S6 [file mmc1.pdf]

iScience, Volume *29*

Supplemental information

Fibrin defines tissue stiffness and biomechanical signaling in regenerating zebrafish hearts as revealed by high-resolution stiffness mapping

Juliane Münch, Tuli Pramanik, Isabell Tunn, Leona Simon, Claudia Jasmin Rödel, Shahrouz Amini, Peter Fratzl, Kerstin Blank, Ondine Cleaver, and Salim Abdelilah-Seyfried

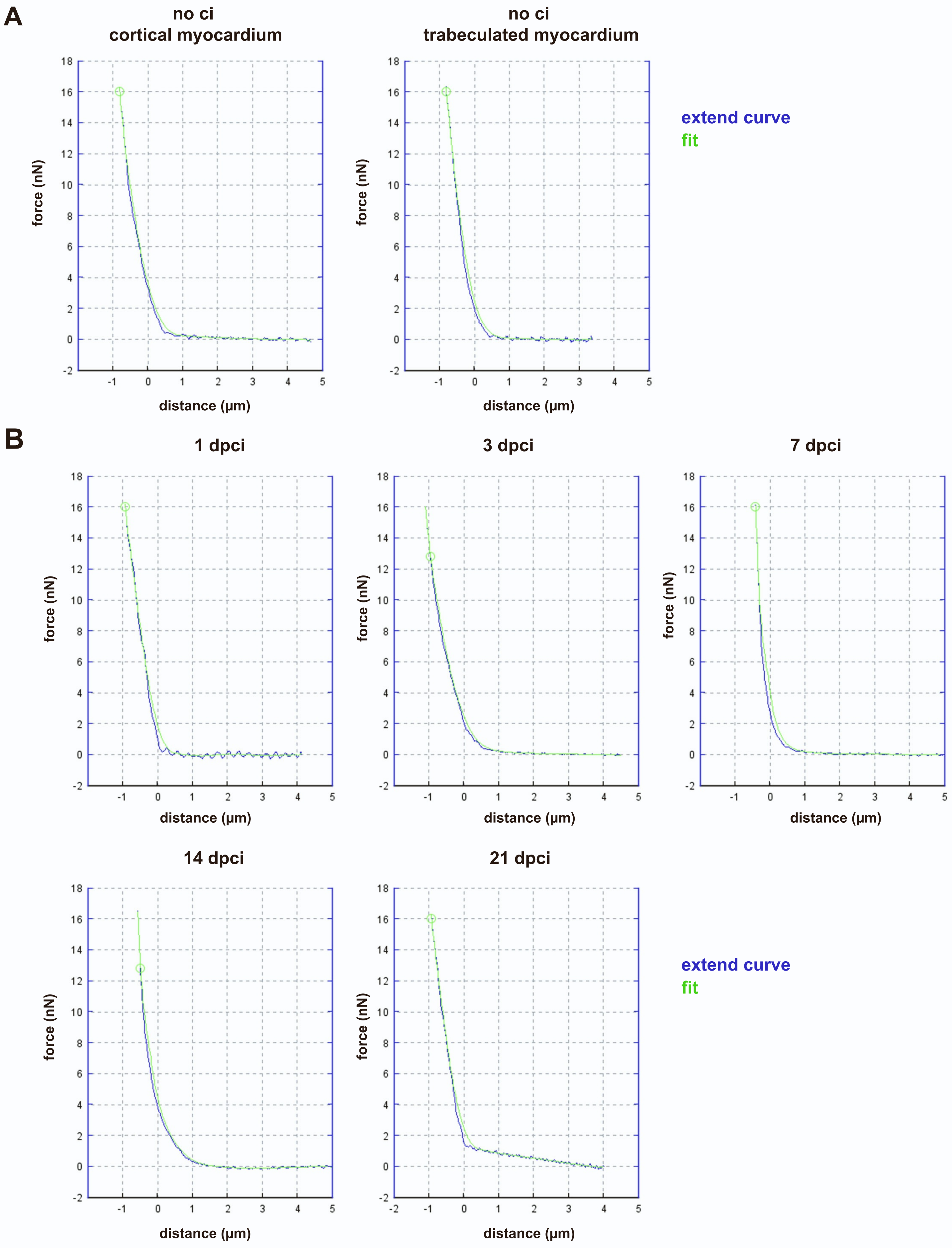

Figure S1. AFM-data analysis. Related to Fiugre 1. A, B Representative force curves (extend) of single AFM measurements on sections of healthy (A) or injured (B) hearts are shown with their corresponding fit using the Hertz-Sneddon model.

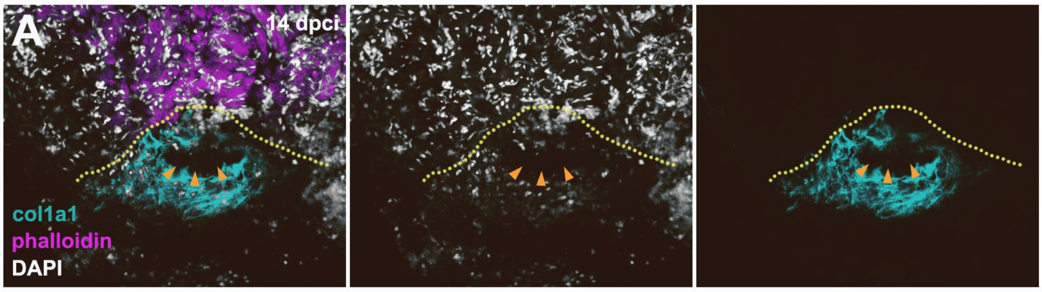

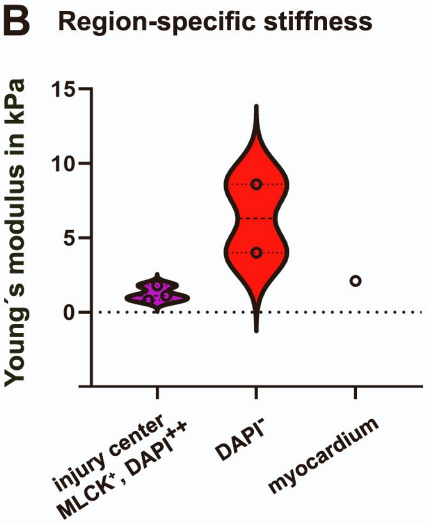

Figure S2. Related to Figure 3. A. Representative confocal image of a heart section (14 dpci) immunostained for Col1a1 and phalloidin.The plotted line demarcates the injury site. Orange arrows indicate the DAPI$^-$ -region. B. Violin plot representing one dataset of Young´s modulus stiffness values of the myosin light chain kinase$^+$ injury center, the DAPI$^-$ -region and the adjacent myocardium. Scale bar: 20µm

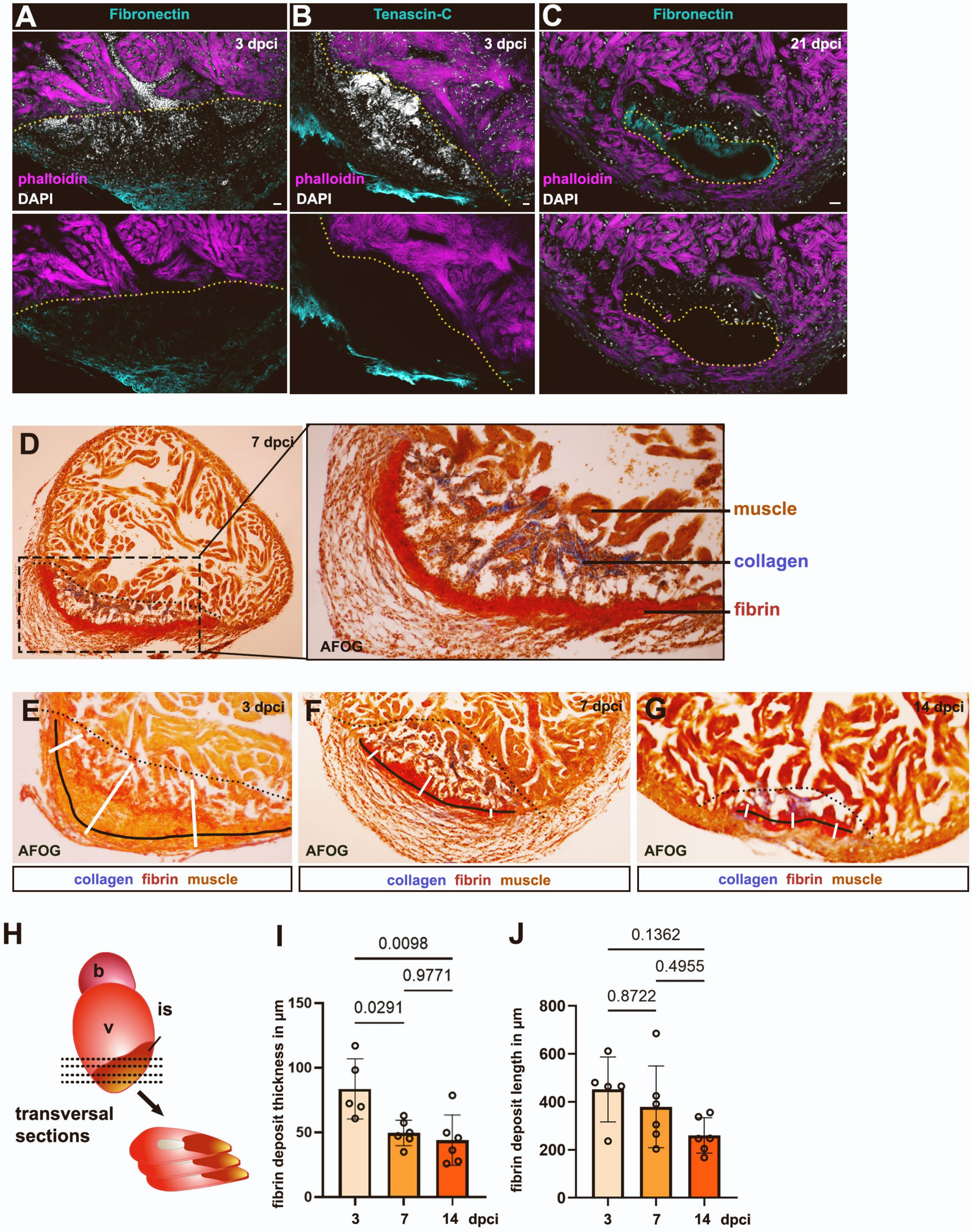

Figure S3. Fibronectin and fibrin deposits at the injury site. Related to Figure 4. A- C. Representative confocal image of a heart section showing Fibronectin deposits at 3 dpci on the injury border and at 21 dpci surrounding the DAPI$^-$ -region or Tenascin-C at the injury border at 3 dpci. D. Acid-Fuchsin-Orange (AFOG) staining of heart sections (7 dpci). Orange: muscle, blue: collagen, red: fibrin. E-G. AFOG staining of heart sections of 3, 7 and 14 dpci. Orange: muscle, blue: collagen, red: fibrin. The black lines indicate the length and white lines the thickness of the injury site. Dotted lines demarcate the injury site. H. Scheme indicating the way of sectioning the cardiac ventricle. I. Bar chart indicating the thickness of fibrin deposits measured on heart sections (E-F, black lines). Individual values and means (bars) +/- SD are represented.JH. Bar chart indicating the length of fibrin deposits measured on heart sections (E-F, white lines). Individual values and means (bars) +/- SD are represented.Ordinary one-way ANOVA with Tukey's multiple comparison test results are indicated. Scale bars: 20μm in C, D, E-G.

A **Genes from GO-term: Fibrin clot formation**

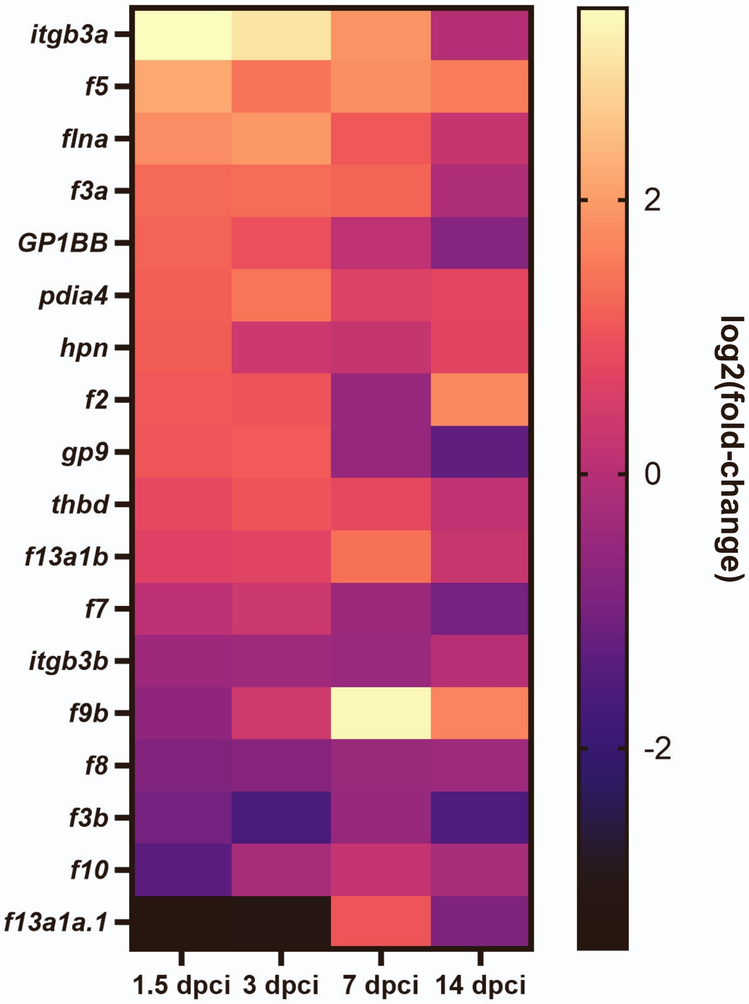

Figure S4. FIbrin clot formation-related genes. Related to Figure 5. A. Heat map indicating expression data from 1.5 – 14 dpci (see Figure 4A) of genes related to the GO-term: “Fibrin clot formation”. Shown is the log2 (fold-change).

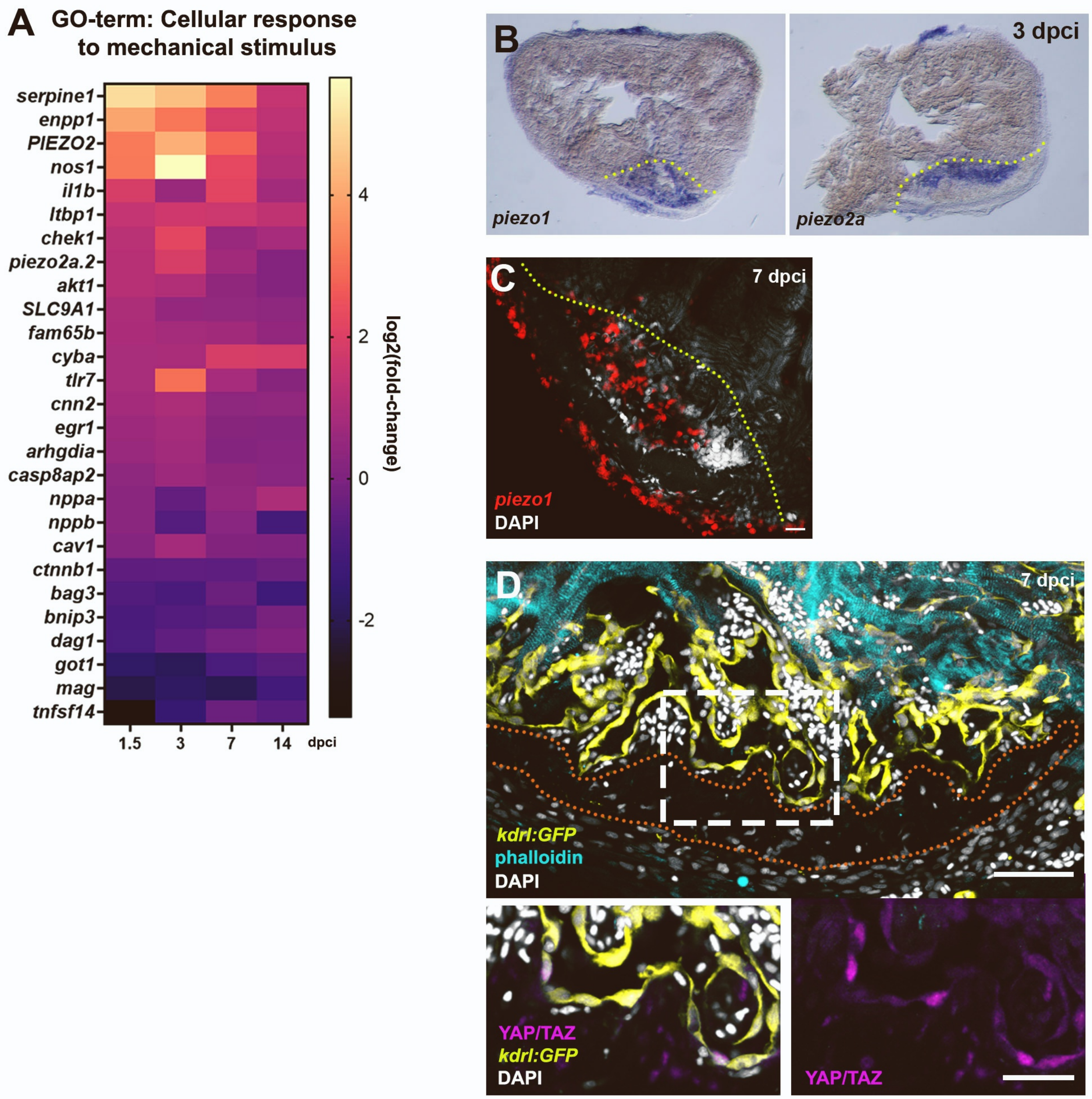

Figure S5. Biomechanical signals in the injured heart. Related to Figure S6. A. Heat map indicating expression data from 1.5 – 14 dpci (see Figure 4A) of genes related to the GO-term: "Cellular response to mechanical stimulus". Represented is the log2 (fold-change). B. In situ hybridization on vibratome heart sections shows *piezo1* and *piezo2a* expression at the injury site (dotted line) at 3 dpci. C. Fluorescent in situ hybridization indicates *piezo1* (red) at the injury center and injury border at 7 dpci. Dotted line demarcates the injury site. D. Immuno-labelled heart section (7 dpci) with endocardial cells (kdrl:GFP, yellow) next to the DAPI- -region (area, marked by the orange dotted line). Some endocardial cells show nuclear YAP/TAZ (magenta, orange arrowheads). 50µm in D, 20µm in C, 10µm in D´.

A

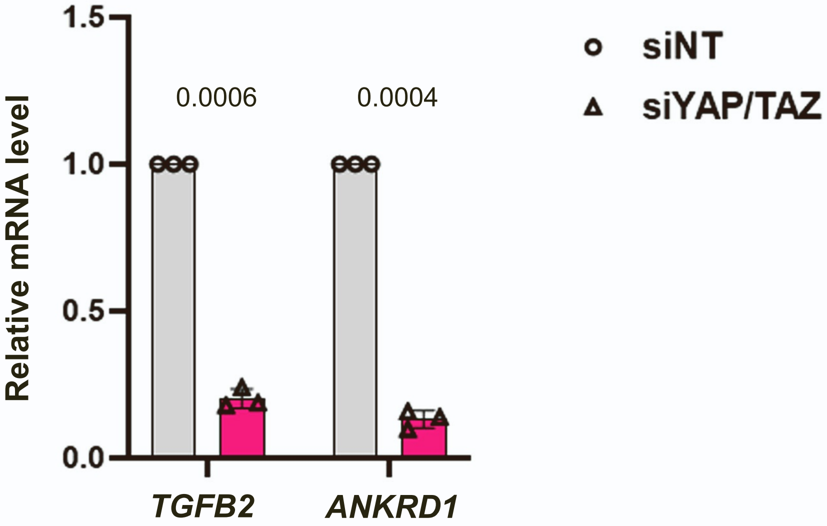

Figure S6. YAP-target gene expression. Related to Fiugre 7. Bar chart indicating r ealative mRNA-levels of ANKRD1 and TGFB2 from HPAECs with siNT or siYAP/TAZ. Individual values and means (bars) +/- SD are represented.One-way ANOVA with Tukey’s multiple comparison test results are indicated.
